# Multi gene mutation signatures in colorectal cancer patients: predict for the diagnosis, pathological classification, staging and prognosis

**DOI:** 10.1186/s12885-021-08108-9

**Published:** 2021-04-09

**Authors:** Yan Zhuang, Hailong Wang, Da Jiang, Ying Li, Lixia Feng, Caijuan Tian, Mingyu Pu, Xiaowei Wang, Jiangyan Zhang, Yuanjing Hu, Pengfei Liu

**Affiliations:** 1 Department of Colorectal Oncology, National Clinical Research Center for Cancer, Key Laboratory of Cancer Prevention and Therapy of Tianjin, Tianjin’s Clinical Research Center for Cancer, Tianjin Medical University Cancer Institute and Hospital, Tianjin, 300060 China; 2 Department of Oncology, Tianjin Academy of Traditional Chinese Medicine Affiliated Hospital, No.354 Beima Road, Hongqiao District, Tianjin, 300120 China; 3 Department of Medical Oncology, The Fourth Hospital of Hebei Medical University, Shijiazhuang, 050000 Hebei China; 4 Department of Nursing, Tianjin Cancer Hospital Airport Hospital, Tianjin, 300300 China; 5 Tianjin Marvel Medical Laboratory, Tianjin Marvelbio Technology Co., Ltd, Tianjin, 300381 China; 6 Tianjin Yunquan Intelligent Technology Co., Ltd, Tianjin, 300381 China; 7 Department of Gynecological Oncology, Tianjin Central Hospital of Obstetrics & Gynecology, No. 156 Nankai Third Road, Nankai District, Tianjin, 300100 China

**Keywords:** Colorectal cancer (CRC), Genotype, Pathological classification, Staging, Prognosis

## Abstract

**Background:**

Identifying gene mutation signatures will enable a better understanding for the occurrence and development of colorectal cancer (CRC), and provide some potential biomarkers for clinical practice. Currently, however, there is still few effective biomarkers for early diagnosis and prognostic judgment in CRC patients. The purpose was to identify novel mutation signatures for the diagnosis and prognosis of CRC.

**Methods:**

Clinical information of 531 CRC patients and their sequencing data were downloaded from TCGA database (training group), and 53 clinical patients were collected and sequenced with targeted next generation sequencing (NGS) technology (validation group). The relationship between the mutation genes and the diagnosis, pathological type, stage and prognosis of CRC were compared to construct signatures for CRC, and then analyzed their relationship with RNA expression, immunocyte infiltration and tumor microenvironment (TME).

**Results:**

Mutations of *TP53*, *APC*, *KRAS*, *BRAF* and *ATM* covered 97.55% of TCGA population and 83.02% validation patients. Moreover, 57.14% validation samples and 22.06% TCGA samples indicated that patients with mucinous adenocarcinoma tended to have *BRAF* mutation, but no *TP53* mutation. Mutations of *TP53*, *PIK3CA*, *FAT4*, *FMN2* and *TRRAP* had a remarkable difference between I-II and III-IV stage patients (*P* < 0.0001). Besides, the combination of *PIK3CA*, *LRP1B*, *FAT4* and *ROS1* formed signatures for the prognosis and survival of CRC patients. The mutations of *TP53*, *APC*, *KRAS*, *BRAF*, *ATM*, *PIK3CA*, *FAT4*, *FMN2*, *TRRAP*, *LRP1B*, and *ROS1* formed the signatures for predicting diagnosis and prognosis of CRC. Among them, mutation of *TP53*, *APC*, *KRAS*, *BRAF*, *ATM*, *PIK3CA*, *FAT4* and *TRRAP* significantly reduced their RNA expression level. Stromal score, immune score and ESTIMATE score were lower in patients with *TP53*, *APC*, *KRAS*, *PIK3CA* mutation compared non-mutation patients. All the 11 gene mutations affected the distributions of immune cells.

**Conclusion:**

This study constructed gene mutation signatures for the diagnosis, treatment and prognosis in CRC, and proved that their mutations affected RNA expression levels, TME and immunocyte infiltration. Our results put forward further insights into the genotype of CRC.

**Supplementary Information:**

The online version contains supplementary material available at 10.1186/s12885-021-08108-9.

## Background

Colorectal cancer (CRC) is one of the most prevalent and lethal malignant diseases worldwide. According to the latest data, the incidence and mortality of CRC ranks 3rd and 2th, respectively, among all global cancer patients [1]. Like most cancers, early diagnosis of CRC contributes to a good prognosis. The 5-year survival for persons with CRC is 64% in the United States. If the disease is detected at an early stage, the 5-year survival rate can be increased to 90% [2]. Simultaneously, pathological classification is also important indicator of prognosis. For example, mucinous colorectal adenocarcinoma is a distinct subtype of CRC, which is more frequently diagnosed in advanced stages and usually has poorer responses to chemotherapy [3]. Tumor microenvironment (TME) plays an important role in tumor development. The synergistic interaction between cancer cells and their supporting cells leads to the malignant tumor. Therefore, TME has a significant effect in cancer patients. At the same time, studies have paid close attention to the effect of immune cells in TME on tumor growth and progression, for example, tumor infiltrating immune cells (TIC) in TME can be used as a promising indicator of therapeutic effect [4]. However, there is still a lack of effective biomarkers for early diagnosis nowadays, and there are few effective prognostic judgment and evaluation indicators for CRC patients.

Genome instability promotes the accumulation of mutations in cancer cells and leads to the rapid evolution of cancer genomes in response to tumor microenvironment and treatment-induced stress. In recent years, data from a large number of genome-wide and exome-wide project have basically elucidated the mutations in most common cancers, and revealed the functional and structural characteristics of cancer genome [5]. Different mutational processes often generate different combinations of mutation types, named as “signatures” [6]. Recently, some studies indicated mutation gene signatures have good performances in predicting the treatment and prognosis in CRC patients [7]. For example, *KRAS*, *BRAF* and *PIK3CA* may have prognostic values in CRC [8], mutation detection of combination of *KRAS*, *BRAF* and *PIK3CA* could contribute to predict the response of CRC patients to EGFR pathway inhibitors [9]. Yu et al. [10] reported that the mutation status of five gene signatures, *CDH10*, *COL6A3*, *SMAD4*, *TMEM132D* and *VCAN*, could predict the survival of CRC patients in two independent cohorts. Ye et al. [11] found that *BRAF* mutation was associated with poor prognosis in Chinese patients receiving anti-EGFR therapy. Furthermore, abundant scientific researches and medical practices have been made and achieved enormous progress about some mutations of them, such as APC, TP53, KRAS, BRAF, PTEN and PIK3CA in the diagnosis, treatment and prognosis of CRC [12, 13]. The NCCN (National Comprehensive Cancer Network) guidelines present that individual genetic test, including *KRAS*, *NRAS*, *BRAF*, etc. can be used for CRC diagnosis and treatment. However, more signatures of gene mutation are still needed to better understand the somatic mutations and genotype of CRC. In addition, studies have shown that gene mutation changes its RNA expression level and immune characteristics, but whether there is an impact in CRC remains unclear.

Incomplete understanding of the genotype is not conducive to accurate treatment. In the present study, we revealed some novel signatures for diagnosis, pathological classification, staging and prognosis of CRC, as well as their correlation with expression level, immune score and distribution of immune cells, in order to give further genetic insights into CRC and provide more comprehensive references for clinical practice.

## Materials and methods

### Cohorts and samples

The overall study included 2 CRC cohorts, the training group (T) and validation group (V). The training group covered 531 patients from 2 The Cancer Genome Atlas projects (TCGAs), namely, The Cancer Genome Atlas Colon Adenocarcinoma (TCGA-COAD, *n* = 397) and Rectal Adenocarcinoma (TCGA-READ, *n* = 134). TCGA-COAD and TCGA-READ mainly study the genetic information of colon adenocarcinoma and rectal adenocarcinoma, respectively. The validation group contained 53 patients, which were collected in department of colorectal oncology of Tianjin Medical University Cancer Institute and Hospital from April 2014 to November 2018.

In training group, all the patients had the tumor tissues and matched normal tissues. The whole genome sequencing (WGS), clinical, and demographic data were downloaded from TCGA (https://tcga-data.nci.nih.gov/docs/publications/tcga/). In the validation group, fresh tumor tissues were sequenced by targeted NGS of the 1000 gene panel (Supplementary Table S1), while 36 paracancerous tissues or 17 leukocytes were used as controls. All patients were followed up by telephone and electronic case, and the follow-up was up to July 20, 2019.

### Genomic DNA extraction

The ≥3 g fresh tissue was collected in tube containing the preservation solution after operation. The genomic DNA was extracted with the Genomic DNA extraction kit (Qiagen, Hilden, Germany) following the manufacturer’s instructions. Afterwards, the DNA samples were purified by Agencourt AMPure XP beads (Agentcourt Biosciences, Beverly, MA, USA).

### Library preparation and targeted next generation sequencing

Library preparation for each sample was performed according to the manufacturer’s protocol. Briefly, ~ 1 μg DNA was randomly sheared into 150–200-base pair fragments using a Covaris M220 instrument (Woburn, MA, USA), followed by library construction with a KAPA Hyper DNA Library Prep Kit (KAPA Biosystems, Wilmington, MA, USA). The adaptor library was amplified and linked, and the total library was accurately quantified by Qubit DNA HS Assay Kit (Invitrogen, CA, USA). A library hybridization kit, SeqCap EZ MedExome Enrichment kits (Roche, Basel, CH), was used to capture target sequences and bead capture and elution hybridization libraries with Roche’s customized 1000 targeted gene probes (Roche, Basel, CH). To construct the targeted gene list, we referred to FoundationOne and Integrated Mutation Profiling of Actionable Cancer Targets (IMPACT), which were designed by two authoritative organizations, Foundation Medicine and Memorial Sloan Kettering Cancer Center (MSK), respectively, and all received FDA approval.

After amplifying the captured library by PCR, the constructed library was sequenced by an Illumina HiSeq Xten sequencer (San Diego, CA, USA). The average sequencing depth of tissue samples was 500 X. It could detect mutations with very low frequency to 0.1%.

### Somatic variant detection

The sequencing data were mapped to the human reference genome (hg19) with Burrows-Wheeler Aligner software for tumor-specific somatic mutation detection. MuTect version 1.1.4 was adopted to process the alignments and identify somatic mutations in tumor tissues compared with the matched normal tissues or leukocytes.

### Mutation signatures

T test or F test in software R (version 3.4.1) was used to detect the correlation between gene variation distribution and each index (cancer species, stage, total survival period, sex, age and race), and the genes with *P* ≤ 0.05 were selected. The ggplot2 (version 2.2.1) was used to display the gene variation distribution of each model.

### Signatures validation

R (version 3.6.1) and IBM SPSS statistics (version 21) were selected for statistical analysis. T-test was used for continuous variables, chi-square test was used for categorical variables, logistic regression was used for cancer classification and staging model validation, and Cox model was used for survival related genes validation. The analysis results were shown using ggplot2 (version3.2.1).

### Tumor microenvironment and immunocyte infiltration

The stromal cells and immune cells in tumor tissue were evaluated by the ESTIMATE algorithm in estimate package in R (version 3.6.1) [14]. The stromal score and immune score were estimated according to the specific biomarkers related to stromal cells and immunocyte infiltration in tumor samples [15]. The stromal scores, immune scores, tumor purity, and estimated scores of each sample were calculated. The higher scores represent higher proportion of corresponding components in TME. Then, the scores and the compositions of 28 kinds of immune cells were compared between different gene mutation states. The data were statistically analyzed with Mann Whitney U test using R (version 3.6.3), *P* < 0.05 was statistically significant.

## Results

### The clinical and sample information

**Fig. 1:**
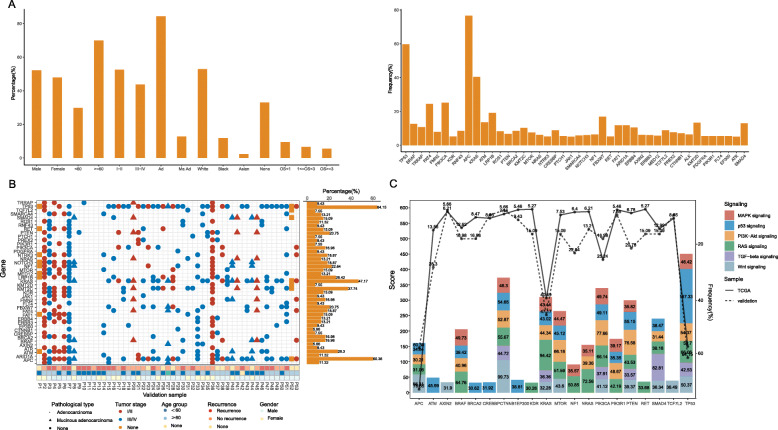
The clinical characteristics and gene mutation landscape in TCGA and validation samples. There are 44 mutation genes occurred and the mutation frequencies were more than 5% both in TCGA and validation samples. **a** clinical information (left) and the mutation frequencies of the 44 genes (right) in TCGA samples; **b** clinical information and the mutation frequencies of the 44 genes in validation samples; **c** the distribution of genes on the pathways over 30 scores in GeneCards

Firstly, we counted the clinical and sample information of patients in the TCGA and validation group. The characteristics of TCGA samples mainly included sex, age, stage, pathological type, race and overall survival (OS) (detailed in Fig. 1a and Supplementary Table S2), which were downloaded directly from TCGA database. While for validation samples, more clinical indicators were involved, such as living habits (drinking, smoking), current medical history (heart disease, hypertension, diabetes, etc.), tumor size and differentiation degree, etc. (detailed in Table 1, Fig. 1b). However, due to race differences, epidemiological trend of diseases, patient’s acceptance and will of gene detection and other reasons, some clinical characteristics of the validation samples were different from those of TCGA. The median age of the TCGA and validation group was 68 and 60, respectively. The TCGA group included 154 III (29.00%) and 78 IV (14.69%) patients, the validation group had 20 III (37.70%) and 16 IV (30.20%) patients. The cohort difference was mainly manifested in patients’ age and tumor stage, and the validation group we collected included more patients < 60 years old (45.28% vs. 29.76%, *P* = 0.019), and more III-IV patients (67.90% vs. 43.69%, *P* = 0.004). The latest large-scale research data both in the United States and Europe revealed that the incidence of CRC is increasing at 40–50 years old, as is the data before 30 years old [16, 17]. The reasons may be related to eating habits, obesity, early screening and so on. Like the United States and some European countries, CRC also has a younger trend in China. Unfortunately, screening in China is not as popular as in the United States and some European countries, and most patients are found to be in the late stage, worse in some rural and remote areas. Therefore, our data were consistent with the incidence trend of CRC and the actual situation in China, and it was reasonable to be used as a validation group.

**Table 1:** The clinical information of validation samples

Index	Classification	Case number	Percentage (%)	Index	Classification	Case number	Percentage (%)
Sex	male	37	69.80	Tumor differentiation status	high	1	1.89
	female	16	30.20		medium	34	64.20
Age	< 60	24	45.28		low	12	22.61
	≥ 60	28	52.83		None	6	11.30
	None	1	1.90	Hypertension	Yes	20	37.70
Tumor stage	I-II	15	28.30		No	31	58.50
	III-IV	36	67.90		None	2	3.80
	None	2	3.80	Diabetes	Yes	5	9.40
Primary diagnosis	Mucinous adenocarcinoma	7	13.20		No	46	86.80
	Other adenocarcinoma	44	83.00		None	2	3.80
	None	2	3.80	Coronary heart disease	Yes	6	11.30
KPS score	80	3	5.70		No	45	84.90
	90	23	43.40		None	2	3.80
	95	12	22.60	Other diseases	Yes	15	28.30
	None	15	28.30		No	36	67.90
Drinking habit	Yes	30	56.60		None	2	3.80
	NO	12	22.60	Therapeutic method	Neoadjuvant chemotherapy + Operation	4	5.70
	None	11	20.80		Operation only	24	45.30
Smoking habit	Yes	24	45.30		Operation + chemotherapy	23	43.40
	NO	23	43.40		Neoadjuvant chemotherapy+Operation+Targeting	2	3.80
	None	6	11.30	OS	< 1 year	17	32.08
Tumor size	< 5 cm	23	43.40		1–3 years	16	30.19
	≥ 5 cm	26	49.10		≥ 3 years	5	9.43
	None	4	7.50		None	5	9.43

OS, survival time; None indicated unclear or unknown

### The gene somatic mutation landscape

In general, we identified 211, 690 mutations, appeared in 18, 298 genes in TCGA cases, and 1, 953 mutations involving 631 genes in validation cases. Furthermore, 44 mutation genes were shared in both cohorts, and they were with frequency of more than 5%. The 44 genes were *TP53*, *APC*, *KRAS*, *BRAF*, *ATM*, *KMT2C*, *LRP1B*, *NF1*, *FBXW7*, *MTOR*, *PTEN*, *PIK3CA*, *RET*, *NTRK3*, *FAT1*, *SMARCA4*, *FAT4*, *SMAD4*, *ARID1A*, *ERBB4*, *BRCA2*, *FMN2*, *AXIN2*, *ERBB3*, *NOTCH3*, *MED12*, *KDR*, *TCF7L2*, *RNF43*, *PREX2*, *CTNNB1*, *ALK*, *NRAS*, *JAK1*, *KMT2D*, *TRRAP*, *CREBBP*, *ROS1*, *PDGFRA*, *PTCH1*, *PIK3R1*, *FLT4*, *EP300* and *ATR.* Among them, both in the two cohorts, the top 3 most frequent genes were *APC*, *TP53* and *KRAS,* and 76.65, 59.70 and 40.49% in TCGA cases, and 60.38, 64.15 and 47.15% in validation cases, respectively. *APC*, the “gatekeeper gene” of CRC, which plays the role of gatekeeper by inducing apoptosis, and also participates in cell migration, adhesion, transcription activation and other processes [18]. About 80% of colorectal adenomas and adenocarcinoma have *APC* gene deletion or inactivation mutations, and the mutation runs through the whole process of carcinogenesis. Approximately half of all CRCs show *TP53* gene mutations, which appear to have little or no prognostic value for CRC patients treated by surgery alone, but are associated with worse survival for patients treated with chemotherapy [19]. It was reported 30–50% of CRC harbor *KRAS* mutations, and *KRAS* mutations in CRC have been associated with poorer survival and increased tumor aggressiveness [20]. Mutations of *APC*, *TP53* and *KRAS* have been proposed as a genetic model, which drives the transition from healthy colonic epithelia to CRC through increasingly dysplastic adenoma, and these mutations lie on alternate pathways of CRC development. In the occurrence and development of CRC, some evidences and theories have been obtained among some of them, such as *FAT4*, *PTCH1*, *ROS1*, *PIK3R1*, *CREBBP*, *FLT4*, *EP300* and *PDGFRA*, and many unanswered and unknown questions are to be discovered [21–25]. Nevertheless, few studies have been reported on *FMN2* (T: 7.91% and V: 16.98%), *TRRAP* (T: 10.73% and V: 9.43%) and *ATR* (T: 5.08% and V: 9.43%) in CRC patients*.* Besides, most of them were on the pathway related to the development of CRC. Figure 1c demonstrated the distribution of these genes on CRC related pathways over 30 scores in GeneCards database (https://www.genecards.org/). The scores represent the correlation between genes and pathways, and this 30 score is the median value after ranking all score values in this study.

In TCGA samples, the frequencies distribution of above 44 genes were listed in Fig. 1a. In addition to *APC*, *TP53* and *KRAS*, frequencies of *FAT4* (24.48%) and *PIK3CA* (25.24%) were also greater than 20%. FAT4, a cadherin-related protein, was shown to function as a tumour suppressor in gastric cancer by modulating Wnt/β-catenin signaling [26]. Mutations in *PIK3CA* play important roles in colorectal carcinogenesis, and are prognosis biomarkers [27]. Overall, these high frequency mutations are the hot spots in CRC. In each validation case, the mutation landscape of them was exhibited in Fig. 1b. Unlike in TCGA case, *TP53* was the gene with the highest mutation frequency, but not APC. Following *APC* and *KRAS*, *KMT2C*, *ATM*, *LRP1B*, *NF1*, *PTEN* and *FBXW7* were ranked in the 4th to 9th places with > 20% mutation frequency. Besides, we found that the mutation frequencies of some genes were very different between the two groups, namely, *PTEN* (6.78% vs. 20.75%, *P* = 0.0004), *APC* (76.65% vs. 60.38%, *P* = 0.009), *ATM* (13.56% vs. 28.30%, *P* = 0.004), *KDR* (5.27% vs. 15.09%, *P* = 0.005), and *FMN2* (7.90% vs. 16.98%, *P* = 0.026). This difference may be due to sample size, age, tumor stage, ethnicity, etc.

### The mutation gene signatures for the diagnosis of CRC

**Fig. 2:**
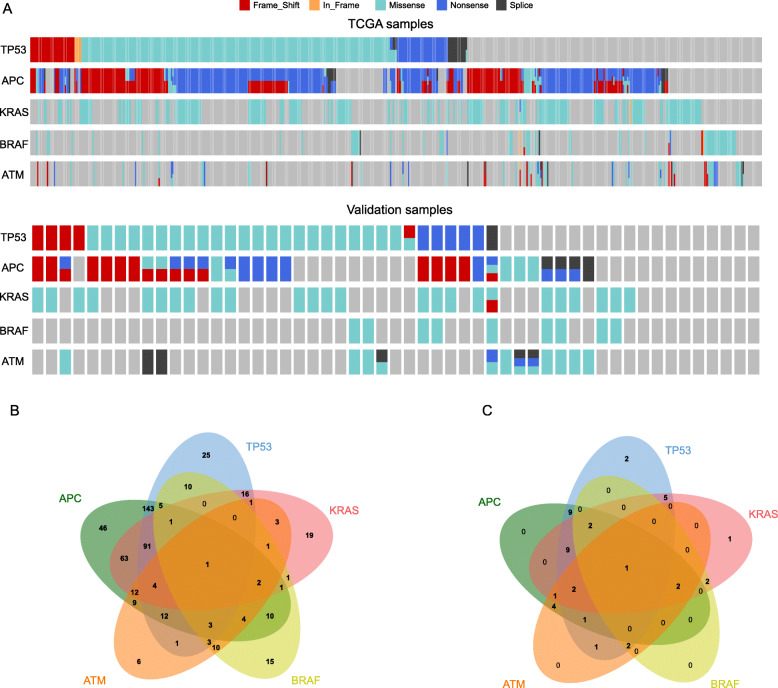
The mutated gene signature for the diagnosis of colorectal cancer. Mutations of *TP53*, *APC*, *KRAS*, *BRAF* and *ATM* covering 97.55% of TCGA population and 84.9% validation patients, and they can be used to distinguish between cancer and paracancer. **a** The mutation distribution of *TP53*, *APC*, *KRAS*, *BRAF* and *ATM* inTCGA and validation patients. Different colors represented different mutation types, and red represented frame-shift mutation, yellow in-frame mutation, green missense mutation, blue nonsense mutation and black splice site mutation; **b** The Venn diagram of mutations of *TP53*, *APC*, *KRAS*, *BRAF* and *ATM* in TCGA population; **c** The Venn diagram of mutations of *TP53*, *APC*, *KRAS*, *BRAF* and *ATM* in validation population

According to the distribution of gene variation in the population, five genes that could cover the largest population were screened, namely, *TP53*, *APC*, *KRAS*, *BRAF* and *ATM.* Mutations of them covered 97.55% (513/518) of TCGA population and 83.02% (44/53) validation patients (Fig. 2a), and can be used to distinguish between cancer and paracancer. Especially when the amount of tissue obtained is small and the pathology is difficult to judge, the signatures can be used to assist diagnosis.

In TCGA cohort, 642 *APC* mutations were detected in 407 cases, including 353 (54.73%) nonsense mutations, 140 frame-shift-del (21.71%), 74 (11.47%) frame-shift-ins, 60 (9.30%) missense, 15 (2.33%) splice-site; 329 *TP53* mutations were detected in 317 cases, including 235 (71.43%) missense mutations, 39 (11.85%) nonsense, 19 frame-shift-del, 14 frame-shift-ins, 15 splice-site, 2 splice-region, 4 in-frame-del and 1 in-frame-ins; 221 *KRAS* mutations were detected in 216 cases, and 219 (99.10%) were missense mutations; 70 *BRAF* mutations were detected in 67 cases, and 87.14% were missense mutations; 104 *ATM* mutations were detected in 73 cases, and 56.73% were missense mutations (Fig. 2a). The Venn graph was showed in Fig. 2b with an online tool (http://bioinformatics.psb.ugent.be/webtools/Venn/). In detail, 27.61% (143/518) of cases were found to contain mutations concurrently in *APC* and *TP53*, 12.16% (63/518) of cases were found to contain mutations concurrently in *APC* and *KRAS*, 17.57% (91/518) mutated in *APC*, *TP53* and *KRAS* in unison, 0.19% (1/518) mutated in *APC*, *TP53*, *KRAS* and *BRAF*, and 0.77% (4/518) mutated in *APC*, *TP53*, *KRAS* and *ATM*.

In validation cohort, 34 cases occurred 35 mutations in *TP53*, and 24 (68.57%) were missenses; 31 cases had 43 mutations in *APC,* and 17 frame-shift, 9 missenses and 13 nonsense; 25 cases appeared 26 mutations in *KRAS*, and 25 (96.15%) were missenses; 9 cases emerged 9 mutations in *BRAF*, and all of them were missenses; 14 cases arose 20 mutations in *ATM*, and 12 (60%) were missenses (Fig. 2a). The Venn graph was showed in Fig. 2c, and the most common combination of mutations was *APC* and *TP53* (20.45%, 9/44), the combination of *APC*, *TP53* and *KRAS*, as well. Next was combinations of *TP53* and *KRAS* (11.36%, 5/44), and *APC* and *ATM* (9.09%, 4/44). The consistency of the combined mutation trend of *APC* and *TP53* in the validation group and TCGA indicated that our verification results were reliable.

Although a gene or a mutation form was different in above two cohorts, mutations in *TP53*, *APC*, *KRAS*, *BRAF* and *ATM* overlapped the most patients and could be used as a diagnostic signature.

### The mutation gene signatures for the pathological classification of CRC

**Fig. 3:**
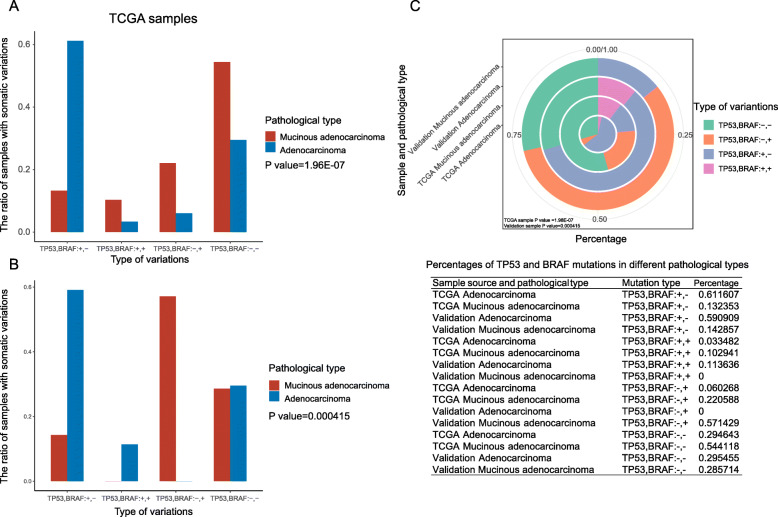
Mutations of *TP53* and *BRAF* were association with clinicopathological characteristics (mucinous adenocarcinoma and other adenocarcinoma). “TP53, BRAF:*+,-*” indicated *TP53* mutated, but *BRAF* not; “TP53, BRAF:*+,+*” indicated both *TP53* and *BRAF* mutated; “TP53, BRAF:*-,+*” indicated *BRAF* mutated, but *TP53* not; “TP53, BRAF:*-,-*” indicated both *TP53* and *BRAF* not mutated. **a** In TCGA samples, “TP53, BRAF:*+,+*”, “TP53, BRAF:*-,+*” and “TP53, BRAF:*-,-*” were more likely to occur in mucinous adenocarcinoma patients (red) than other adenocarcinoma patients (blue). P represents the significance of the overall difference between the four groups. **b** In validation samples, more than 50% mucinous adenocarcinoma patients were “TP53, BRAF*:-,+*”, while none of them were other adenocarcinoma. P represents the significance of the overall difference between the four groups. **c** The circular map and data chart of *TP53* and *BRAF* mutation percentages in different pathological types. The outer to inner ring represents the mucinous adenocarcinoma in the validation group, other adenocarcinoma in the validation group, mucinous adenocarcinoma in the TCGA group, and other adenocarcinoma in the TCGA group

More than 95% of CRC was adenocarcinoma, and mucinous adenocarcinoma accounts for about 10% of all cases with a poor outcome [28]. In TCGA group, 68 (12.80%) cases were mucinous adenocarcinoma, and 448 (84.37%) were other adenocarcinoma. There were 189 (35.59%) right colon cancer cases and 247 (46.52%) left colon cancer cases. In validation cohort, there were 51 (96.23%) adenocarcinoma, including 7 mucinous adenocarcinoma and accounting for 13.21% in all cases. We found that mutations of *TP53* and *BRAF* were significantly related to the pathological types of cancer in both cohorts (Fig. 3, *P* < 0.0001 and = 0.0004, respectively).

In TCGA cases, 54.41% mucinous adenocarcinoma cases were non-mutated both in *TP53* (−) and *BRAF* (−), and 61.16% other adenocarcinoma cases were *TP53* mutated (+) and *BRAF* (−) (Fig. 3a and c)*.* Besides, the group of *TP53* (+) and *BRAF* (+) tended to mucinous adenocarcinoma, as well as group of *TP53* (−) and *BRAF* (+) (Fig. 3a and c). Our research found that regardless of *TP53* status, *BRAF* mutations were more common in mucinous adenocarcinoma. This finding was consistent with previous conclusions.

In validation cases, 59.09% other adenocarcinoma were *TP53* (+) and *BRAF* (−), which was similar to those in TCGA cases (Fig. 3b and c). However, unlike results in TCGA cases, all *TP53* (−) and *BRAF* (+) cases were mucinous adenocarcinoma, and all *TP53* (+) and *BRAF* (+) cases were other adenocarcinoma, and *TP53* (−) and *BRAF* (−) cases had no obvious difference in different pathological types (Fig. 3b and c). The reasons for the above differences may be related to the different race, age and stage distributions between the two groups, and the small number of samples in the validation group may also be one of reasons. In addition, we used logistic regression analysis in validation cases to get the coefficient of the pathological classification = 3.192 × (mutation of *TP53*) - 2.954 × (mutation of *BRAF*) + 1.493, and mutation of *TP53* or *BRAF =* 0 (mutated) or 1 (no mutated); and mucinous adenocarcinoma (the coefficient = 0) and other adenocarcinoma (the coefficient = 1). The accuracy rate of this model was 94.1% for the whole, 100% for other adenocarcinoma, and 57.1% for mucinous type. In summary, mutation detection of *TP53* and *BRAF* can be used for pathological classification of CRC.

### The mutation gene signatures for the tumor stage of CRC

**Fig. 4:**
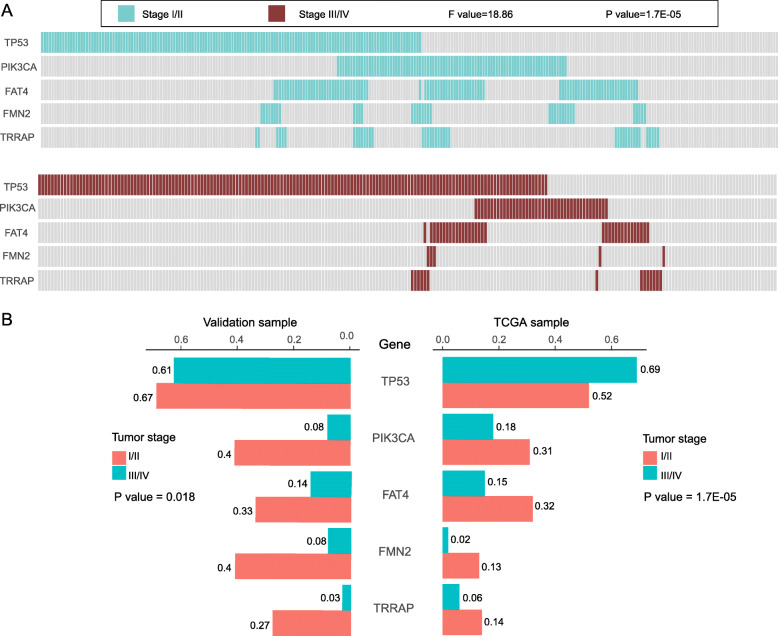
The mutated gene signature for the stage of colorectal cancer. Mutations of *TP53*, *PIK3CA*, *FAT4*, *FMN2* and *TRRAP* had a remarkable difference between I-II and III-IV stage patients (*P* < 0.0001). **a** The distribution of the 5 mutated genes in different stages of TCGA samples. The figures above and below were stage I-II group and stage III-IV group, respectively. Each bar represents a patient. **b** The gene mutation frequencies of *TP53*, *PIK3CA*, *FAT4*, *FMN2* and *TRRAP* in validation (left) and TCGA (right) samples

Tumor staging is an important diagnostic index, which is contribute to guide treatment and prompt prognosis. Here, we divided the samples into stage I-II and stage III-IV groups. In the discovery queue, after counting all possible combinations, the signature of 5 genes was obtained which was closely related to the tumor stage with the minimum *P* value, namely *TP53*, *PIK3CA*, *FAT4*, *FMN2* and *TRRAP* (F = 18.86 and *P* < 0.0001, Fig. 4a, b). It was further confirmed in the validation queue (*P* = 0.018, Fig. 4b). Moreover, both in the discovery and validation queues, *PIK3CA*, *FAT4*, *FMN2* and *TRRAP* had higher mutation frequencies in I-II stage group (Fig. 4b). Interestingly, *TP53* mutation was contradictory between two cohorts in different stages, with higher frequency in stage III-IV group in discovery queue, and no remarkable difference between stage I-II and stage III-IV groups in validation queue (Fig. 4b). Besides, the logistic regression analysis was also performed to obtain the coefficient of the stage = 0.003 × (mutation of *TP53*) - 1.937 × (mutation of *PIK3CA*) - 0.468 × (mutation of *FAT4*) - 1.245 × (mutation of *FMN2*) - 1.573 × (mutation of *TRRAP*) + 1.790: mutation of gene = 0 (mutated) or 1 (no mutated); and I or II stage (the coefficient = 0) and III or IV (the coefficient = 1). The accuracy rate of the mutation signature composed of *TP53*, *PIK3CA*, *FAT4*, *FMN2* and *TRRAP* was 80.4% overall, 53.3% for I-II stage, and 91.7% for III-IV stage. Therefore, mutation detection of *TP53*, *PIK3CA*, *FAT4*, *FMN2* and *TRRAP* could help to identify the CRC stage.

### The mutation gene signatures for the survival of CRC

**Fig. 5:**
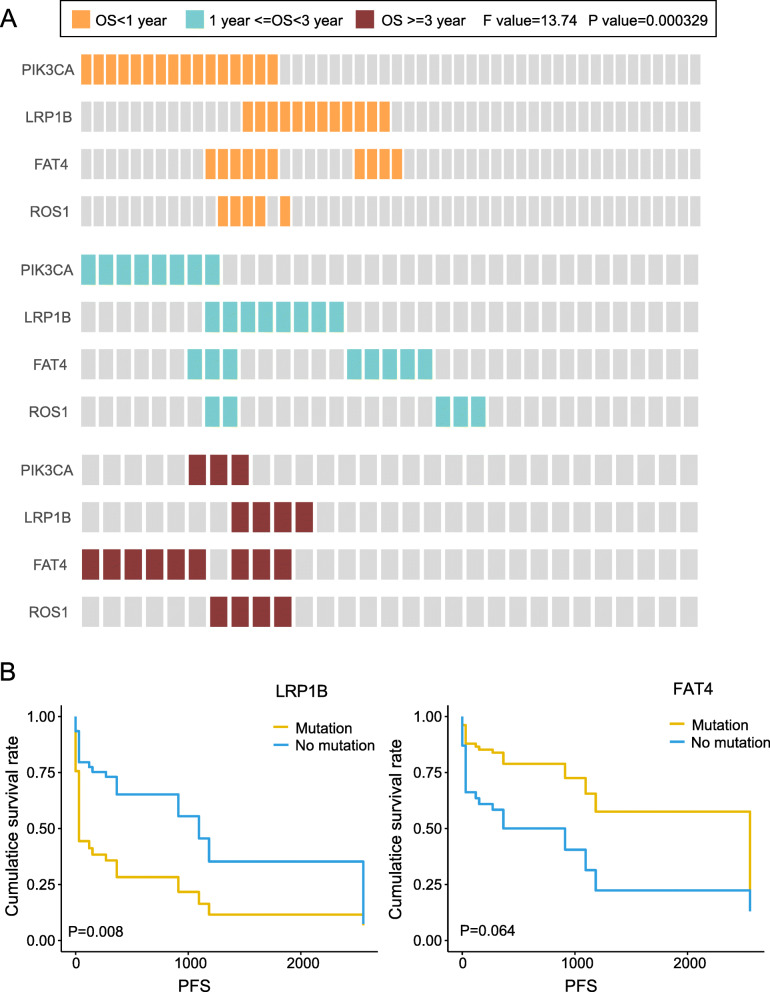
The mutated gene signature for the prognosis of colorectal cancer. *PIK3CA, LRP1B, FAT4* and *ROS1* were significantly correlated with overall survival (*P* < 0.001). **a** In TCGA samples, the distribution of the 4 mutated genes in patients with different OS. The figures from top to bottom were OS < 1 year group, 1 < OS < 3 years group, and OS > 3 years group in sequence. **b** In validation samples, mutation survival curve of *LRP1B* (left) and *FAT4* (right) genes

In the discovery cohort, samples were firstly divided into three groups according to the OS (< 1 year, *n* = 50; 1–3 years, *n* = 35; and > 3 years, *n* = 29). After multiple combinations of the above 44 genes, we found that the mutation signature of *PIK3CA* (25.24%), *LRP1B* (19.21%), *FAT4* (24.48%) and *ROS1* (8.29%) showed the most significant difference among the above three groups (F = 13.74 and *P* = 0.0003, Fig. 5a). In our clinical samples, the above result was not authenticated. Yet, we found some other interesting results: mutation of *LRP1B* portended to a higher of recurrence and shorter progression-free survival (PFS); mutation of *FAT4* portended to a lower of recurrence and longer PFS (Fig. 5b). Therefore, we can detect the mutation of *PIK3CA*, *LRP1B*, *FAT4* and *ROS* to predict the survival of CRC patients.

Taken together, we screened 11 genes (*TP53*, *APC*, *KRAS*, *BRAF*, *ATM*, *PIK3CA*, *FAT4*, *FMN2*, *TRRAP*, *LRP1B* and *ROS1*) associated with diagnose, pathological classification, tumor stage and survival of CRC, and mutation detection of these genes can be applicable in the clinic of CRC.

### Correlation analysis between the CRC-related gene mutations and RNA expression levels in the TCGA cohort

**Fig. 6:**
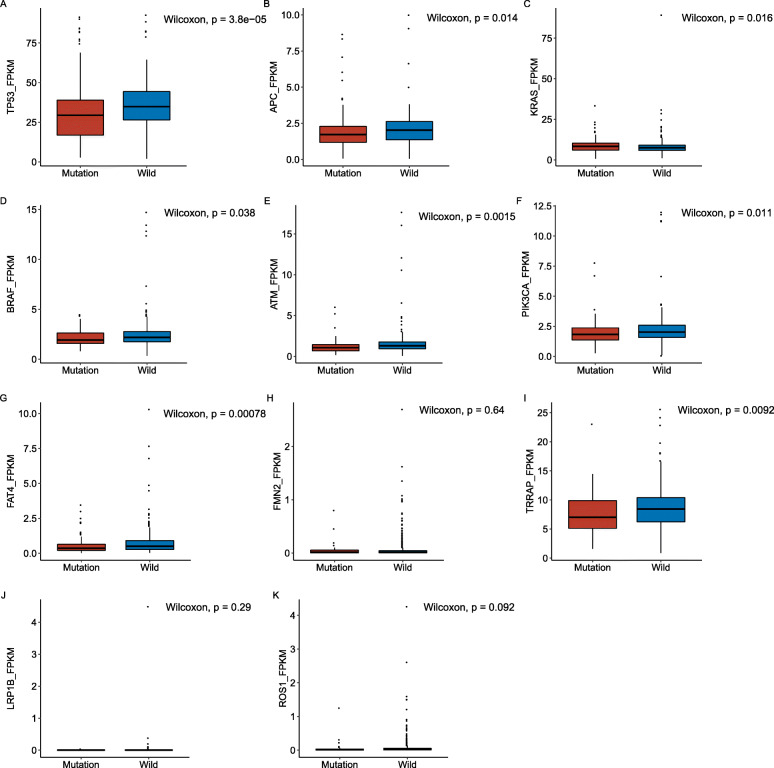
The relationship between gene mutation and its RNA expression level in TCGA cohort. Mutations of *TP53*, *APC*, *KRAS*, *BRAF*, *ATM*, *PIK3CA*, *FAT4*, and *TRRAP* significantly down-regulated their RNA expression levels (*P* < 0.001). **a-k** Comparison of RNA expression levels between mutation and non-mutation gene of *TP53* (**a**), *APC* (**b**), *KRAS* (**c**), *BRAF* (**d**), *ATM* (E), *PIK3CA* (**f**), *FAT4* (**g**), *FMN2* (**h**), *TRRAP* (**i**), *LRP1B* (**j**) and *ROS1* (**k**) in CRC patients

To identify the effect of the CRC-related gene mutations above on gene expressions, we conducted the correlation analysis between the mutation data and expression data in each screened gene above from the TCGA cohort. Among the 11 screened genes (*TP53*, *APC*, *KRAS*, *BRAF*, *ATM*, *PIK3CA*, *FAT4*, *FMN2*, *TRRAP*, *LRP1B* and *ROS1*), the mutation of 8 genes (except *FMN2*, *LRP1B* and *ROS1*) significantly down-regulated their own expression level (*P* < 0.05, Fig. 6). Therefore, this preliminary result proved that the mutations of *TP53*, *APC*, *KRAS*, *BRAF*, *ATM*, *PIK3CA*, *FAT4* and *TRRAP* functioned as negative factors to affect their transcriptions.

### Correlation analysis between the CRC-related gene mutations and immune microenvironment

**Fig. 7:**
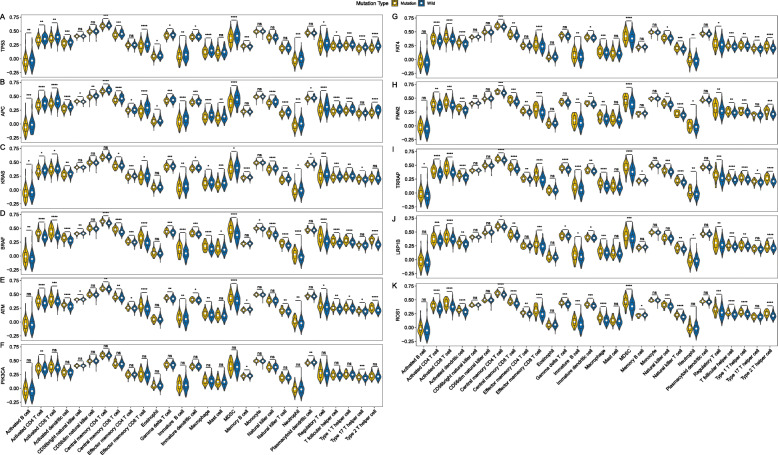
Relationship between immunocyte infiltration and gene mutation status in the mutated gene signatures. Activated CD4 T cell and T-follicular helper cell had significant differences in all genes between mutated and non-mutated status (*P* < 0.05), while the distribution of CD56dim natural killer cell, monocyte and eosinophil were different in less genes. **a-k** Comparison of distribution of immune cells between mutation and non-mutation gene of *TP53* (**a**), *APC* (**b**), *KRAS* (**c**), *BRAF* (**d**), *ATM* (**e**), *PIK3CA* (**f**), *FAT4* (**g**), *FMN2* (**h**), *TRRAP* (**i**), *LRP1B* (**j**), and *ROS1* (**k**) in CRC patients

**Fig. 8:**
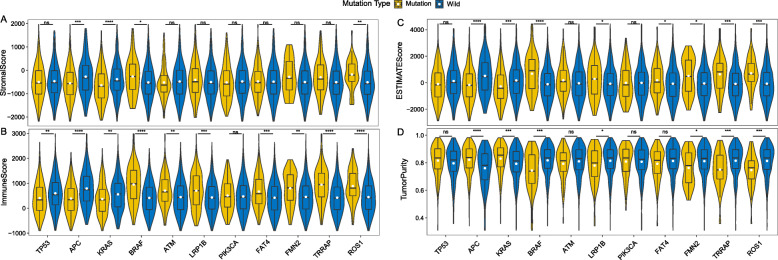
The relationship between tumor microenvironment (TME) and gene mutation status in the mutated gene signatures. Patients with mutant *TP53*, *APC*, *KRAS*, *PIK3CA* had lower stromal score, immune score, ESTIMATE core and higher tumor purity compared with non-mutated patients, and the TME in patients with *BRAF*, *ATM*, *LRP1B*, *FAT4*, *FMN2*, *TRRAP*, and *ROS1* mutations showed the opposite trend. **a** Comparison of stromal score between mutated and non-mutated patients. **b** Comparison of immune score between mutated and non-mutated patients. **c** Comparison of ESTIMATE score between mutated and non-mutated patients. **d** Comparison of tumor purity between mutated and non-mutated patients

Crosstalk between cancer cells and immune microenvironment is essential to the tumor development. To identify whether the screened genes in CRC involved the immune microenvironment, we conducted a correlative analysis between the CRC-related gene mutations and immune microenvironment. Based on the gene expression data from the TCGA cohort, we calculated the abundance of 28 immune cells in the TME. Through the correlation analysis, we found that most of the cells had significant differences between the mutated and non-mutated states of the 11 screened genes (Fig. 7). Among them, activated CD4 T cell and T-follicular helper cell had significant differences in all genes between mutated and non-mutated status (*P* < 0.05), while the distribution of CD56dim natural killer cell, monocyte and eosinophil were different in less genes (Fig. 7). TME includes stromal cells, tumor cells, and immune cells. The higher the stromal score and immune score, the lower the purity of tumor. Figure 8 showed the TME in CRC patients between mutation and non-mutation genes. Among all genes, stromal score, immune score, and estimate core of mutant *TP53*, *APC*, *KRAS*, *PIK3CA* were lower (Fig. 8a-c), and tumor purity was relatively high (Fig. 8d). In contrast, mutant *BRAF*, *ATM*, *LRP1B*, *FAT4*, *FMN2*, *TRRAP*, and *ROS1* had higher stromal score (except ATM), immune score and ESTIMATE score, and the tumor purity was lower than the wild-type (Fig. 8).

## Discussion

In this report, we have identified numerous somatic mutations of CRC both in TCGA and validation cohorts, and then extended four mutation signatures for the diagnosis, pathological classification, tumor stage, and survival time, including 11 screened genes in total. These data improved the current understanding of the molecular type of CRC and facilitated clinical molecular diagnosis and prognosis prediction. Mutations of *TP53*, *APC*, *KRAS*, *BRAF* and *ATM* were a diagnosis signature for CRC; *TP53* and *BRAF*, a mutation signature for the pathological classification; *TP53*, *PIK3CA*, *FAT4*, *FMN2* and *TRRAP*, a mutation signature for tumor stage; *PIK3CA*, *LRP1B*, *FAT4* and *ROS1*, a mutation signature for prognosis.

First and foremost, 44 shared mutated genes were identified in both cohorts with frequency of more than 5%. Most of them have been studied in CRC and possessed similar frequencies in the 2 cohorts, suggesting these mutation genes are reliable. More specifically, it is basically formed a consensus that CRC progression is sequential gains of mutations in Wnt signaling, RAS signaling, TGF-beta signaling, p53 signaling, PI3K-Akt signaling and MAPK signaling [29]. After annotation with GeneCards database, many of them were found to be involved in the pathways related to CRC (Fig. 1c, Supplementary Fig. 1 and Table S3): *CTNNB1*, *APC*, *TP53*, *MTOR*, PIK3CA and *PTEN*, etc. were enriched in Wnt signaling; *KRAS*, *NRAS*, *PIK3CA*, *TP53*, *BRAF* and *CTNNB1*, etc. in RAS signaling; *SMAD4*, *CTNNB1*, *TP53*, *PIK3CA*, *KRAS* and *PTEN* in TGF-beta signaling; *TP53*, *PTEN*, *CTNNB1*, *PIK3CA*, *ATM* and *MTOR*, etc. in p53 signaling; *PIK3CA*, *PTEN*, *MTOR*, *TP53*, *CTNNB1* and *PIK3R1*, etc. in PI3K-Akt signaling; *PIK3CA*, *BRAF*, *TP53*, *CTNNB1*, *MTOR* and *KRAS*, etc. in MAPK signaling. Additionally, *PIK3CA*, *KRAS*, *CTNNB1*, *TP53* and *PTEN* are simultaneously in all of the 6 pathways listed above. This result further indicated that the 44 mutated genes we screened were meaningful and worthy of further study. This result demonstrated the importance of gene detection for high frequency mutated genes and cross genes in various pathways related to CRC from another level.

Next, we firstly discovered the mutation combination of *TP53* (T: 59.70% and V: 64.15%), *APC* (T: 76.65% and V: 60.38%), *KRAS* (T: 40.49% and V: 47.17%), *BRAF* (T: 12.62% and V: 16.98%) and *ATM* (T: 13.56%and V: 28.30%) can overlap the most patients, 97.55% of TCGA population and 84.9% validation patients (Fig. 2a). A similar study reported a biochip assaying 28 mutations in the *KRAS*, *BRAF*, *TP53*, and *APC* genes for detection of CRC, and 71% cancerous tissues were covered [30]. However, the forerunner’s study did not brought *ATM* into and had a lower coverage. As discussed in the foregoing, *TP53*, *APC*, *KRAS* were the most common and popular genes, and were prone to mutation in CRC. A previous study about it reported that the progressive acquisition of activating or loss of them drives the adenoma to carcinoma transition. They also revealed that the most common combination of mutations was *TP53* and *APC* (27.1%), and only 6.6% concurrently contained mutations of *TP53*, *APC*, and *KRAS* [31]. In our study, *TP53* and *APC* were confirmed again to be the most common mutation combination with 27.61 and 20.45% in TCGA and validation cohorts, respectively. Whereas, the frequency of mutations of *TP53*, *APC*, *KRAS* in union was much higher than the previous study, 17.57% in TCGA cases, and even higher in validation cases (20.45%). The reason for this difference may be related to the race and disease history of the enrolled patients. The previous patients were white and had no history of previous cancer or diverticular disease; while the TCGA patients contained black and Asian and the validation patients were Asian, and both of them had no restrictions on the history of disease. As for *BRAF*, about 10% of CRC patients are characterized by a mutation in *BRAF* gene resulting in a valine-to-glutamate change at the residue 600 (V600E) [32]. *BRAF* inhibitors have been developed and benefited melanoma patients. Although, CRC patients do not respond much efficiently, and most patients at the end of the track ultimately developed resistance to these inhibitors. However, in the phase III BEACON CRC trial, encorafenib (BRAF inhibitor), binimetinib (MEK inhibitor), and cetuximab (anti-EGFR monoclonal antibody) significantly improved the OS and ORR of patients with *BRAF* V600E mutant mCRC compared to the current standard chemotherapy, and its safety is consistent with the known safety of each drug [33]. Moreover, *BRAF*-mutated tumors are often right sided, more recurrent in woman, higher grade, and associated with microsatellite instability (MSI) and old age [34]. In our results, the *BRAF*-mutated frequencies were separately 12.62 and 16.98% in TCGA and validation cohort, and higher in the elderly and less in mucinous adenocarcinoma (*P* < 0.05). Less in-depth researches have made about *ATM* mutation, and it may be associated with PARP inhibitors and EGFR-targeted therapies [35, 36]. Thus, few studies have brought *ATM* mutation into CRC signatures. In present study, we found the mutation *ATM* (T: 13.56%and V: 28.30%) made the mutation signature of *TP53* (T: 59.70% and V: 64.15%), *APC* (T: 76.65% and V: 60.38%), *KRAS* (T: 40.49% and V: 47.17%), *BRAF* (T: 12.62% and V: 16.98%) more extensive and coverage than the other genes among the other 39 genes.

The pathological classification and tumor stage are essential indexes of the clinical diagnosis, and important references for therapeutic schedule, and they also can predict the prognosis to some extent. Hence, understanding their relationship with gene mutation may contribute to better prognosis and treatment of the disease. It was reported that *KRAS* gene was often mutated in colorectal adenocarcinoma, and 88.9% (8/9) of *KRAS* mutated cases were stage III or IV diseases [37]. We did not find a correlation between *KRAS* mutations and adenocarcinoma, probably because the patients we enrolled were basically adenocarcinoma, except for a few unclear ones. Our data also did not find that *KRAS* mutation was related to III-IV stage, which may be related to the difference stages of recruitment. Patients in III-IV stage of the previous studies were less (7.14%, 9/126), and those were many more in our study (70.59 and 43.69% in validation and TCGA cases). Thus, our results were more credible. Colorectal mucinous adenocarcinoma is a subtype of CRC with prominent mucin production associated with advanced stage at diagnosis, and *BRAF* mutation [38]. In our study, we also proved mucinous adenocarcinoma tended to have *BRAF* mutation. More importantly, we further revealed mucinous adenocarcinoma and other adenocarcinoma were also related to the *TP53* mutation, and mutations of *TP53* and *BRAF* were considered as a signature to distinguish pathological types, which greatly complements and refines the previous studies (detailed in Fig. 3). In the same way, we firstly identified *TP53*, *PIK3CA*, *FAT4*, *FMN2* and *TRRAP* as a mutation signature for tumor stage (Fig. 4). As expounded in the previous article, mutations of *TP53*, *PIK3CA*, *FAT4* have a relatively clear impact and relevance on tumor staging [19, 26, 27]. However, few reports were conducted about *FMN2* and *TRRAP* in CRC. Here we firstly incorporated them into the tumor stage, and the signature composed of *TP53*, *PIK3CA*, *FAT4*, *FMN2* and *TRRAP* had the accuracy rate of 80.4% overall, and 91.7% for III-IV stage. Besides, we used the logistic regression model to analyze the above two signatures and got the regression model. According to the model formula, we can better understand the positive-negative role and weight of each gene in the signature.

The prognosis of CRC is also an important topic in clinical and academic research. Survival time, recurrence, metastasis, and death are common prognostic factors. In TCGA cohort, we selected *PIK3CA*, *LRP1B*, *FAT4* and *ROS1* as a mutation signature for prognosis (Fig. 5a). At present, the relevant research is mainly focused on the single or two gene combination mutations for the study of prognosis, multiple gene combined mutations were not found in the study of prognosis. Pietrantonio F et al. [39] excavated *ALK*, *ROS1*, and *NTRK* rearrangements define a new and rare subtype of mCRC with extremely poor prognosis. Compared it, the signature of our result was with wider application. It is well known *PIK3CA* mutation is predictive of poor survival in patients with CRC [40]. Less evidences have been obtained to support mutation of *LRP1B* and *FAT4* are associated with the prognosis of CRC. Interestingly, we found mutation of *LRP1B* portended to a higher recurrence and shorter PFS; mutation of *FAT4* portended to a lower recurrence and longer PFS (Fig. 5b). Unfortunately, the predicted survival effect of the signature was not fully verified in the validation group. It is suspected that the signature needs further research and confirmation, which is what we will do in the future.

For the above 11 mutated genes that significantly associated with diagnosis, pathological type, stage and survival, we further analyzed the relationship between the mutation status and RNA expression level, immune invasion and TME. With the development of multi-omics analysis, much more attentions have been paid to the study of mutation effects, such as the expression of downstream genes of candidate mutations in regulatory networks, the relationship between gene mutations and changes of RNA expression levels, or the impact of mutations on pathways. Previous studies have shown that some mutations introduce stop codons prematurely and reduce mRNA transcripts, and some of them affect protein activity by changing amino acid sequences [41]. In this study, mutations of *TP53*, *APC*, *KRAS*, *BRAF*, *ATM*, *PIK3CA*, *FAT4* and *TRRAP* resulted in a significant decrease in their RNA expression level in TCGA cohort (*P* < 0.05). However, due to the lack of transcriptome sequencing data in clinical cohort, the results are unable to verify, and the mechanism of these mutations down regulating RNA expression level and its impact on CRC were not clear. Therefore, large prospective experiments should be conducted in the future to investigate the mechanism of how mutations work.

The distribution of immune cells, stromal scores, immune scores and ESTIMATE scores were significantly different between wild-type and mutant of the 11 genes, and were used to verify the accuracy of these mutation signatures. Subtle changes in the distribution of immune cells may have different effects on tumor progression [42]. In this study, some wild-type genes showed higher immunocyte infiltration, while others showed lower immunocyte infiltration or no statistical difference (Fig. 7). The stromal score and immune score were based on specific biomarkers associated with stromal and immunocyte infiltration in tumor samples. Stromal and immune scores form the ESTIMATE score, and are negatively correlated with tumor purity [43]. In this study, mutant *TP53*, *APC*, *KRAS*, and *PIK3CA* had lower stromal score, immune score, ESTIMATE score and higher tumor purity (Fig. 8). These scores of all genes were consistent with the distribution of immune cells, that is, the mutant genes with lower immunocyte infiltration also had lower immune scores. However, in this study, there was no significant correlation between immunocyte infiltration and RNA expression levels, which needs to be further confirmed and verified in an independent cohort. In addition, the relationship among the expression level of some checkpoints and immunocyte infiltration and TME is also the focus of future research.

In this study, there were two main limitations. First, the availability of data is limited by public resources. The MSI and MSS data that are important to evaluate the CRC patients, cannot get in this study. And some important clinical characteristics were also missing, such as therapeutic regimen, transcriptome sequencing data and expression of immune checkpoints. Second, the small sample number in validation can only prevail the general hypothesis of this study, and the clinical significance of the selected signatures cannot be verified. Therefore, this study need a multi-data analysis and further verification to support the views in the future.

## Conclusions

In conclusion, we have revealed gene mutations and identified 11 gene signatures for the diagnosis, pathological classification, staging and prognosis in CRC, and proved that mutation of most of these signatures significantly reduced their RNA expression, and affected the TME and immunocyte infiltration. This study puts forward further insights into the genotype and therapy of CRC, and is contribute to the personalized diagnosis and treatment.

## Supplementary Information

**Additional file 1.**

**Additional file 2.**

## Data Availability

The datasets used and/or analyzed during the current study are available from the corresponding author on reasonable request.

## Abbreviations

CRC: Colorectal cancer
NGS: Next-generation sequencing
WGS: Whole genome sequencing
OS: Overall survival
PFS: Progression-free survival
MSI: microsatellite instability
