# Supplementary material for: Multi gene mutation signatures in colorectal cancer patients: predict for the diagnosis, pathological classification, staging and prognosis
Source: BMC Cancer. 2021 Apr 9;21:380. doi: 10.1186/s12885-021-08108-9 (PMC8034139; doi:10.1186/s12885-021-08108-9)
Supplement: Supplementary file 1 — Additional file 1. [file 12885_2021_8108_MOESM1_ESM.docx]

**Supplementary Table S1. List of 1,000 genes related to carcinogenesis and development**

| ABCB1 | ABL2 | ABCF2 | ACE | ACER2 | ACOT11 | ACPP |
| --- | --- | --- | --- | --- | --- | --- |
| ACSL1 | ACSM5 | ACSS3 | ACTL6B | ADAM23 | ADAM33 | ADAMTS12 |
| ADAMTS16 | ADAMTS19 | ADAMTS20 | ADAMTS5 | ADAMTSL1 | ADD2 | AGMAT |
| AGTPBP1 | AHCTF1 | AK5 | AKR1B10 | AKR1C1 | AKT1 | AKT2 |
| AKT3 | ALDH1A3 | ALDH2 | ALG5 | ALK | ALX4 | AMOT |
| ANK2 | ANKRD13D | ANKRD20A4 | ANKRD27 | ANKRD28 | ANKRD30A | ANKRD30B |
| ANKRD36B | ANO2 | AP1B1 | AP1G2 | AP3B1 | APAF1 | APC |
| APLP2 | APMAP | APPL2 | AQP12A | AR | ARAF | ARFGAP1 |
| ARFRP1 | ARHGAP35 | ARHGAP40 | ARHGEF1 | ARHGEF7 | ARNTL | ASH2L |
| ASTN1 | ASXL2 | ATAD2B | ATIC | ATM | ATP10B | ATP10D |
| ATP12A | ATP2C1 | ATP6V0A2 | ATP8B2 | ATR | ATXN2 | ATXN7L2 |
| AURKA | AURKB | AXL | BAP1 | BAX | BBS9 | BCAS1 |
| BCAS2 | BCL2 | BMPR1B | BRAF | BRCA1 | BRCA2 | BRD2 |
| BRD3 | BRD4 | BRF1 | BRWD3 | BSG | BTK | BTNL3 |
| BTRC | C11orf30 | C12orf5 | C19orf38 | C1orf112 | C1orf35 | C1QA |
| C1S | C20orf112 | C2orf47 | C2orf62 | C7orf53 | C8orf34 | C9orf43 |
| CACNA1A | CACNA1D | CACNA1E | CADM2 | CAMKK1 | CAPRIN1 | CARS |
| CARS2 | CASC4 | CASP8 | CASP8AP2 | CASQ2 | CATSPER2 | CBFB |
| CBL | CBR3 | CCDC155 | CCDC159 | CCDC17 | CCND1 | CCND2 |
| CCND3 | CCNE1 | CCT3 | CCT6B | CD1E | CD274 | CD300LF |
| CD5L | CD9 | CD97 | CDA | CDH1 | CDH18 | CDH24 |
| CDH26 | CDK11A | CDK12 | CDK13 | CDK14 | CDK18 | CDK19 |
| CDK4 | CDK6 | CDK8 | CDKN1A | CDKN1B | CDKN2A | CDKN2B |
| CDS1 | CEACAM20 | CECR2 | CELA2B | CGN | CHD3 | CHD4 |
| CHD6 | CHEK1 | CHEK2 | CISD3 | CLCN7 | CLEC16A | CLINT1 |
| CNGB3 | CNKSR2 | CNOT3 | CNOT4 | CNTN1 | CNTN4 | CNTN5 |
| CNTNAP3B | CNTNAP5 | COASY | COL14A1 | COL16A1 | COL19A1 | COL1A1 |
| COL25A1 | COL4A5 | COL4A6 | COL5A1 | COL5A2 | COL5A3 | COL6A5 |
| COL6A6 | COL9A1 | COPA | COPG1 | CPA1 | CPSF3 | CPSF6 |
| CRKL | CRTAM | CRTAP | CRYBG3 | CSF1R | CSMD1 | CSMD3 |
| CSN3 | CSNK1E | CSPP1 | CTCF | CTIF | CTNNA2 | CTNNB1 |
| CTSF | CYP2A13 | CYP3A4 | CYP4A11 | CYTH4 | DCLK2 | DCST1 |
| DDB1 | DDR1 | DDR2 | DDX3X | DEPDC4 | DGKK | DHCR24 |
| DHDDS | DHX9 | DIAPH1 | DKC1 | DLST | DMD | DMXL1 |
| DMXL2 | DNAH10 | DNAH5 | DNAH9 | DNAJC11 | DNAJC9 | DNMT3A |
| DOCK11 | DOCK3 | DOT1L | DPP10 | DPRD | DRGX | DUOX1 |
| DYSF | DZANK1 | ECHDC1 | EDN1 | EEF1A1 | EFCAB5 | EFCAB6 |
| EFCAB7 | EFHA2 | EFNA5 | EGFR | EIF2B5 | EIF2C2 | EIF3E |
| EIF3I | EIF4ENIF1 | EIF4H | ELAVL3 | ELL3 | EMID2 | ENPP2 |
| ENTPD6 | EPB41L2 | EPB41L4B | EPHA2 | EPHA3 | EPHA5 | EPHB1 |
| ERBB2 | ERBB3 | ERBB4 | ERCC1 | ERG | ESR1 | ETV6 |
| EXOC4 | EXOC5 | EXOC6 | EXOC7 | EXTL3 | EYA4 | EZH2 |
| F8 | F9 | FAH | FAM114A2 | FAM131B | FAM135B | FAM13C |
| FAM157B | FAM177B | FAM21A | FAM3A | FAM49A | FAM49B | FAM5C |
| FAM86B1 | FAN1 | FANCC | FASTK | FAT1 | FBXW7 | FCGR2A |
| FCGR2B | FCGR3A | FDCSP | FGFR1 | FGFR2 | FGFR3 | FGFR4 |
| FLCN | FLNC | FLOT2 | FLT1 | FLT3 | FLT3LG | FLT4 |
| FMN2 | FMNL3 | FNDC4 | FNIP2 | FOLH1 | FOXA1 | FOXJ2 |
| FOXL2 | FRG2B | FRMD4A | FRMPD2 | FRMPD4 | FSD2 | FSHR |
| FUBP1 | FUNDC1 | GAB2 | GAB3 | GABRD | GAD2 | GALNT13 |
| GALNT14 | GATA3 | GFRAL | GIGYF1 | GINS4 | GIPR | GKN2 |
| GLB1L3 | GLYR1 | GMDS | GNA11 | GNAQ | GNAS | GNPTAB |
| GOLGA4 | GPAT2 | GPATCH2 | GPR114 | GPR125 | GPR133 | GPR144 |
| GPS2 | GRIA3 | GRIK2 | GSTP1 | GUCY2C | GYLTL1B | HAAO |
| HAP1 | HAUS5 | HAUS6 | HCN1 | HDAC1 | HDAC4 | HDAC6 |
| HEATR7B2 | HECTD4 | HECW1 | HECW2 | HGF | HIST1H3B | HLA-DRB1 |
| HLA-DRB5 | HMCN1 | HMHA1 | HNF4A | HOMER2 | HPS3 | HPS4 |
| HRAS | HSPA12B | HSPD1 | HYDIN | IBSP | IDH1 | IDH2 |
| IFT172 | IGF1R | IGSF9 | IKBKAP | IKBKE | IL11RA | IL13RA2 |
| IL1RAPL1 | IL27RA | IL7R | IMPG1 | INHBA | INPP4B | INPP5J |
| IQCA1 | IRS2 | ITFG2 | ITGA8 | ITGA9 | ITIH1 | ITLN2 |
| ITM2A | ITPKB | ITPR1 | JAK1 | JAK2 | JAK3 | KCNAB2 |
| KCNH6 | KCNQ2 | KDM4A | KDM6A | KDR | KEAP1 | KIAA0195 |
| KIAA0226 | KIAA0319 | KIAA0922 | KIAA1191 | KIAA1199 | KIAA1211L | KIF13A |
| KIF1B | KIF26B | KIFAP3 | KIFC1 | KIR2DL3 | KIR3DL3 | KIT |
| KLHL1 | KLHL14 | KLK1 | KMT2B | KMT2C | KRAS | KRT2 |
| KRT9 | KRTAP5-5 | KTN1 | L3MBTL1 | LARP1 | LCN10 | LCT |
| LCTL | LETM1 | LGALS13 | LILRB3 | LILRB4 | LIPN | LMAN1L |
| LMBR1L | LPCAT4 | LPHN3 | LRBA | LRP1B | LRP2 | LRP4 |
| LRRC16B | LRRC2 | LRRC7 | LRRC72 | LRRD1 | LRRFIP2 | LRSAM1 |
| LTBP1 | LUC7L2 | LUZP4 | MAEL | MAGI1 | MAN2A1 | MAP2K1 |
| MAP2K2 | MAP2K4 | MAP3K1 | MAP4K1 | MAPK1 | MAPK3 | MAPKAPK3 |
| MAPRE3 | MAST1 | MBIP | MBTPS2 | MCF2L2 | MCL1 | MCOLN2 |
| MDGA2 | MDM2 | MDM4 | MDN1 | MED12 | MED23 | MEFV |
| MET | METTL14 | METTL5 | MGAM | MICALL1 | MID1 | MIER2 |
| MITF | MLH1 | MLH3 | MLL | MLPH | MORC1 | MORN1 |
| MPL | MRPL1 | MRPL24 | MRPS18B | MS4A1 | MSH2 | MSH3 |
| MSH6 | MSI1 | MTA2 | MTHFR | MTOR | MTR | MTRR |
| MUC5B | MUS81 | MYB | MYBPC2 | MYC | MYCBP2 | MYD88 |
| MYH15 | MYH2 | MYH4 | MYH8 | MYH9 | MYL5 | MYL6 |
| MYLK2 | MYO3A | MYOD1 | NACAD | NARF | NAT10 | NAV3 |
| NBPF1 | NBPF10 | NCF2 | NCKAP1 | NCOR1 | NCOR2 | NEK5 |
| NELL1 | NF1 | NF2 | NFE2L2 | NIPBL | NLGN3 | NLRC3 |
| NLRP4 | NMI | NOP2 | NOS1 | NOS2 | NOTCH1 | NOTCH2 |
| NOTCH3 | NOTCH4 | NRAS | NRXN1 | NRXN2 | NT5C3L | NTM |
| NTRK1 | NTRK3 | NUDCD2 | NUP205 | NUP210 | NUTM1 | NWD1 |
| NXF1 | NXF5 | OBP2A | OBP2B | OCA2 | ODZ3 | OR2T4 |
| OR4A15 | OR4C6 | OR5L2 | OR6F1 | OSBPL10 | OTOA | OTOGL |
| OVCH1 | P4HB | PABPC4 | PACS2 | PAEP | PAGE1 | PALB2 |
| PARK2 | PARP4 | PCK2 | PCLO | PCNT | PCNXL2 | PCSK5 |
| PCYT1A | PDCD6 | PDE1C | PDE2A | PDE4DIP | PDGFRA | PDGFRB |
| PDIA5 | PDILT | PDK1 | PDRG1 | PEX6 | PGAP1 | PHACTR3 |
| PHF20L1 | PHYH | PI4KB | PIK3CA | PIK3CB | PIK3R1 | PIK3R2 |
| PIP4K2C | PIP5K1C | PIWIL1 | PKD1L2 | PKHD1 | PKLR | PLAC8 |
| PLCB4 | PLCZ1 | PLEC | PLK2 | PLOD3 | PLXNA1 | PMS1 |
| PMS2 | POLDIP2 | POLE | POLR2J | POLR3B | POLR3GL | POLRMT |
| POM121L12 | POTEG | PPA1 | PPDPF | PPEF1 | PPFIBP2 | PPIL2 |
| PPP1R17 | PPP4R4 | PQBP1 | PREB | PREX2 | PRKAA1 | PRKACA |
| PRKAG3 | PRKCD | PRKDC | PRKX | PRRX1 | PRSS1 | PRUNE |
| PSG2 | PSG5 | PSIP1 | PSMB1 | PSMB5 | PSMC4 | PSMC6 |
| PSTPIP1 | PTBP3 | PTCD3 | PTCH1 | PTCH2 | PTEN | PTGS2 |
| PTGES3L-AARSD1 | PTPLAD1 | PTPN11 | PTPN13 | PTPRA | PTPRD | PTPRM |
| PYHIN1 | QRICH2 | RAB1B | RAB3GAP2 | RAB6A | RAC2 | RAF1 |
| RALBP1 | RAPGEF2 | RARA | RARB | RASEF | RB1 | RBM6 |
| RBMX | RCC1 | REC8 | REG1B | RELN | RERE | RET |
| RFWD2 | RFX3 | RHEB | RHOA | RICTOR | RNF215 | RNF219 |
| RNF43 | ROCK1 | ROS1 | RPL22 | RPL36A | RPS5 | RPS6KA1 |
| RPS6KB1 | RPTOR | RPUSD4 | RREB1 | RRM1 | RUNDC3A | RUNX1 |
| RYR2 | RYR3 | SAFB2 | SAG | SAGE1 | SAMD8 | SCN10A |
| SCN3A | SCN7A | SCN9A | SDK2 | SEC14L4 | SEC24B | SEH1L |
| SELP | SEMA6A | 12-Sep | SERPINA7 | SETD1B | SETD2 | SF1 |
| SF3B1 | SF3B14 | SF3B3 | SGCZ | SGIP1 | SGK1 | SGPL1 |
| SH2D3A | SH3BGR | SH3PXD2A | SHISA4 | SI | SIDT2 | SIK3 |
| SIM1 | SIM2 | SLC13A3 | SLC17A6 | SLC17A8 | SLC25A1 | SLC25A30 |
| SLC26A3 | SLC2A2 | SLC30A5 | SLC35B2 | SLC35B4 | SLC38A4 | SLC38A5 |
| SLC43A1 | SLC45A1 | SLC4A10 | SLC4A4 | SLC5A1 | SLC6A5 | SLC8A1 |
| SLCO1B7 | SLIT1 | SMARCA4 | SMARCB1 | SMO | SMTN | SNTG1 |
| SOD2 | SPAG16 | SPATA13 | SPG20 | SPINT1 | SPPL2A | SPPL3 |
| SPRED1 | SPTA1 | SRC | SRRT | SSBP3 | SSH2 | SSPO |
| ST18 | ST6GALNAC1 | STAG2 | STAT1 | STAT3 | STAT4 | STAT6 |
| STK11 | STK11IP | STK31 | STX3 | SULT1A4 | SUPT5H | SUPT6H |
| SYCP2L | SYK | SYNE1 | SYNE2 | SYNJ2 | TAF1B | TAF6 |
| TARBP1 | TBC1D1 | TBC1D21 | TBC1D3 | TBC1D5 | TBL1X | TBP |
| TBX15 | TBX22 | TBX3 | TCF20 | TCF4 | TCP10 | TCP11 |
| TEK | TERT | TESC | TEX35 | TFDP1 | TGDS | TGM2 |
| TGM5 | THBS2 | THEM5 | THOC1 | THSD7A | THSD7B | TIMD4 |
| TIMM44 | TIMP3 | TJP3 | TLE1 | TLL1 | TMC2 | TMED8 |
| TMEM104 | TMEM120B | TMEM132D | TMEM145 | TMEM247 | TMEM80 | TMEM87A |
| TMPRSS2 | TMTC4 | TMX3 | TNFAIP6 | TNFSF4 | TNN | TNNT1 |
| TNR | TNS3 | TOP1 | TP53 | TP53BP1 | TPCN1 | TPH2 |
| TPMT | TPTE | TRIM33 | TRIM51 | TRIM58 | TRIML1 | TRIO |
| TRIP11 | TRMT112 | TRPC5 | TRUB1 | TSC1 | TSC2 | TSGA10 |
| TSKS | TSPAN12 | TSR2 | TTF2 | TTLL3 | TTN | TUBA3C |
| TUBGCP4 | TUBGCP5 | TYK2 | TYRP1 | U2AF1 | U2AF2 | UBASH3A |
| UBE2Q1 | UBE4B | UCHL3 | UCK2 | UGT1A1 | ULK3 | UMPS |
| UNC13A | UNC13D | UNC5D | USP12 | USP34 | USP39 | USP45 |
| USP48 | VAV1 | VEGFA | VEZF1 | VHL | VILL | VIT |
| VPS13A | VPS33B | VSIG4 | WAS | WASL | WDR44 | WDR52 |
| WDR62 | WDR66 | WDR72 | WDTC1 | WLS | WSCD2 | WWP2 |
| XBP1 | XPC | XPO4 | XPO5 | XRCC1 | ZAP70 | ZBTB8OS |
| ZC3H13 | ZC3H7B | ZDHHC11 | ZFC3H1 | ZFR | ZMYM4 | ZNF143 |
| ZNF350 | ZNF385A | ZNF414 | ZNF512B | ZNF541 | ZNF563 | ZNF614 |
| ZNF687 | ZNF705B | ZNF705G | ZNF711 | ZNF804B | ZSWIM8 |  |

**Supplementary Table S2. Clinical data of the 531 patients obtained from TCGA database**

| **Sub_id** | **Pro_id** | **Primary**  **diagnosis** | **Sex** | **Race** | **Vital**  **status** | **Days to death** | **Tumor stage** | **Age at**  **diagnosis (d)** | **Tissue or organ of origin** |
| --- | --- | --- | --- | --- | --- | --- | --- | --- | --- |
| TCGA-3L-AA1B | TCGA-COAD | Adenocarcinoma, NOS | female | black or african american | alive | -- | stage i | 22379 | Cecum |
| TCGA-4N-A93T | TCGA-COAD | Adenocarcinoma, NOS | male | black or african american | alive | -- | stage iiib | 24523 | Ascending colon |
| TCGA-4T-AA8H | TCGA-COAD | Mucinous adenocarcinoma | female | black or african american | alive | -- | stage iia | 15494 | Descending colon |
| TCGA-5M-AAT4 | TCGA-COAD | Adenocarcinoma, NOS | male | black or african american | dead | 49 | stage iv | 27095 | Ascending colon |
| TCGA-5M-AAT6 | TCGA-COAD | Adenocarcinoma, NOS | female | black or african american | dead | 290 | stage iv | 14852 | Ascending colon |
| TCGA-5M-AATE | TCGA-COAD | Adenocarcinoma, NOS | male | black or african american | alive | -- | stage iia | 27870 | Ascending colon |
| TCGA-A6-2671 | TCGA-COAD | Adenocarcinoma, NOS | male | white | dead | 1331 | stage iv | 31329 | Sigmoid colon |
| TCGA-A6-2672 | TCGA-COAD | Adenocarcinoma, NOS | female | white | alive | -- | stage iiib | 30237 | Colon, NOS |
| TCGA-A6-2674 | TCGA-COAD | Mucinous adenocarcinoma | male | white | alive | -- | stage iv | 26292 | Sigmoid colon |
| TCGA-A6-2675 | TCGA-COAD | Adenocarcinoma, NOS | male | white | alive | -- | stage iia | 28813 | Sigmoid colon |
| TCGA-A6-2677 | TCGA-COAD | Adenocarcinoma, NOS | female | white | dead | 740 | stage iiic | 25143 | Colon, NOS |
| TCGA-A6-2679 | TCGA-COAD | Adenocarcinoma, NOS | female | white | alive | -- | stage iib | 26820 | Ascending colon |
| TCGA-A6-2680 | TCGA-COAD | Adenocarcinoma, NOS | female | black or african american | alive | -- | stage ii | 26533 | Hepatic flexure of colon |
| TCGA-A6-2681 | TCGA-COAD | Adenocarcinoma, NOS | female | white | alive | -- | stage iia | 26929 | Colon, NOS |
| TCGA-A6-2682 | TCGA-COAD | Adenocarcinoma, NOS | male | white | dead | 424 | stage iv | 25683 | Colon, NOS |
| TCGA-A6-2684 | TCGA-COAD | Adenocarcinoma, NOS | female | white | alive | -- | stage i | 27708 | Colon, NOS |
| TCGA-A6-2685 | TCGA-COAD | Adenocarcinoma, NOS | female | black or african american | alive | -- | stage iia | 17757 | Colon, NOS |
| TCGA-A6-2686 | TCGA-COAD | Adenocarcinoma, NOS | female | white | dead | 1126 | stage iia | 29623 | Colon, NOS |
| TCGA-A6-3807 | TCGA-COAD | Adenocarcinoma, NOS | female | white | alive | -- | stage iiic | 19624 | Sigmoid colon |
| TCGA-A6-3808 | TCGA-COAD | Adenocarcinoma, NOS | male | white | alive | -- | stage iia | 26666 | Colon, NOS |
| TCGA-A6-3809 | TCGA-COAD | Mucinous adenocarcinoma | female | white | alive | -- | stage iib | 26274 | Colon, NOS |
| TCGA-A6-3810 | TCGA-COAD | Adenocarcinoma, NOS | male | white | alive | -- | stage iia | 22999 | Colon, NOS |
| TCGA-A6-4105 | TCGA-COAD | Adenocarcinoma, NOS | male | white | dead | 442 | stage iia | 29097 | Ascending colon |
| TCGA-A6-4107 | TCGA-COAD | Adenocarcinoma, NOS | female | white | alive | -- | stage iiib | 21064 | Ascending colon |
| TCGA-A6-5656 | TCGA-COAD | Adenocarcinoma, NOS | male | white | alive | -- | stage i | 27184 | Sigmoid colon |
| TCGA-A6-5657 | TCGA-COAD | Adenocarcinoma, NOS | male | black or african american | alive | -- | stage iiib | 23920 | Colon, NOS |
| TCGA-A6-5659 | TCGA-COAD | Adenocarcinoma, NOS | male | white | alive | -- | stage i | 30028 | Colon, NOS |
| TCGA-A6-5660 | TCGA-COAD | Adenocarcinoma, NOS | male | black or african american | alive | -- | stage iiic | 26803 | Colon, NOS |
| TCGA-A6-5661 | TCGA-COAD | Adenocarcinoma, NOS | female | black or african american | alive | -- | stage iia | 29446 | Ascending colon |
| TCGA-A6-5664 | TCGA-COAD | Adenocarcinoma, NOS | male | black or african american | alive | -- | stage iiic | 29426 | Colon, NOS |
| TCGA-A6-5665 | TCGA-COAD | Adenocarcinoma, NOS | female | white | alive | -- | stage iia | 30915 | Ascending colon |
| TCGA-A6-5666 | TCGA-COAD | Adenocarcinoma, NOS | male | white | alive | -- | stage iic | 28514 | Colon, NOS |
| TCGA-A6-5667 | TCGA-COAD | Adenocarcinoma, NOS | female | white | alive | -- | stage iiib | 14756 | Sigmoid colon |
| TCGA-A6-6137 | TCGA-COAD | Adenocarcinoma, NOS | male | white | alive | -- | stage iiib | 20095 | Hepatic flexure of colon |
| TCGA-A6-6138 | TCGA-COAD | Adenocarcinoma, NOS | male | white | alive | -- | stage i | 22523 | Cecum |
| TCGA-A6-6142 | TCGA-COAD | Adenocarcinoma, NOS | female | white | alive | -- | stage iva | 20485 | Sigmoid colon |
| TCGA-A6-6648 | TCGA-COAD | Adenocarcinoma, NOS | male | white | alive | -- | stage iva | 20641 | Descending colon |
| TCGA-A6-6649 | TCGA-COAD | Adenocarcinoma, NOS | male | white | alive | -- | stage iiib | 24237 | Ascending colon |
| TCGA-A6-6650 | TCGA-COAD | Adenocarcinoma, NOS | female | black or african american | alive | -- | stage iia | 25516 | Ascending colon |
| TCGA-A6-6651 | TCGA-COAD | Adenocarcinoma, NOS | female | white | alive | -- | stage iiib | 20110 | Colon, NOS |
| TCGA-A6-6652 | TCGA-COAD | Adenocarcinoma, NOS | male | black or african american | alive | -- | stage iva | 21638 | Sigmoid colon |
| TCGA-A6-6653 | TCGA-COAD | Adenocarcinoma, NOS | male | white | alive | -- | stage i | 29996 | Colon, NOS |
| TCGA-A6-6654 | TCGA-COAD | Adenocarcinoma, NOS | female | white | alive | -- | stage iiib | 23953 | Colon, NOS |
| TCGA-A6-6780 | TCGA-COAD | Mucinous adenocarcinoma | male | white | alive | -- | stage iia | 27250 | Ascending colon |
| TCGA-A6-6781 | TCGA-COAD | Mucinous adenocarcinoma | male | white | alive | -- | stage iiic | 15842 | Colon, NOS |
| TCGA-A6-6782 | TCGA-COAD | Adenocarcinoma, NOS | male | white | alive | -- | stage iib | 30012 | Ascending colon |
| TCGA-A6-A565 | TCGA-COAD | Mucinous adenocarcinoma | female | black or african american | dead | 494 | stage iiic | 12526 | Transverse colon |
| TCGA-A6-A566 | TCGA-COAD | Mucinous adenocarcinoma | female | black or african american | dead | 758 | stage iiib | 20181 | Descending colon |
| TCGA-A6-A567 | TCGA-COAD | Adenocarcinoma, NOS | male | black or african american | dead | 1881 | stage iv | 20493 | Sigmoid colon |
| TCGA-A6-A56B | TCGA-COAD | Adenocarcinoma, NOS | male | black or african american | dead | 1711 | stage iiib | 20903 | Sigmoid colon |
| TCGA-AA-3488 | TCGA-COAD | Adenocarcinoma, NOS | male | not reported | dead | 153 | stage iv | 21519 | Rectosigmoid junction |
| TCGA-AA-3489 | TCGA-COAD | Adenocarcinoma, NOS | male | not reported | dead | 214 | stage ii | 27606 | Colon, NOS |
| TCGA-AA-3492 | TCGA-COAD | Adenocarcinoma, NOS | female | not reported | dead | 92 | stage ii | 32871 | Ascending colon |
| TCGA-AA-3494 | TCGA-COAD | Adenocarcinoma, NOS | male | not reported | alive | -- | stage iv | 20089 | Colon, NOS |
| TCGA-AA-3495 | TCGA-COAD | Adenocarcinoma, NOS | male | not reported | alive | -- | stage i | 28886 | Hepatic flexure of colon |
| TCGA-AA-3496 | TCGA-COAD | Adenocarcinoma, NOS | female | not reported | alive | -- | stage ii | 30438 | Ascending colon |
| TCGA-AA-3502 | TCGA-COAD | Adenocarcinoma, NOS | male | not reported | alive | -- | stage i | 26724 | Colon, NOS |
| TCGA-AA-3506 | TCGA-COAD | Adenocarcinoma, NOS | male | not reported | alive | -- | stage i | 28275 | Colon, NOS |
| TCGA-AA-3509 | TCGA-COAD | Adenocarcinoma, NOS | female | not reported | alive | -- | stage ii | 19786 | Sigmoid colon |
| TCGA-AA-3510 | TCGA-COAD | Adenocarcinoma, NOS | male | not reported | alive | -- | stage ii | 25902 | Colon, NOS |
| TCGA-AA-3511 | TCGA-COAD | Adenocarcinoma, NOS | male | not reported | alive | -- | stage ii | 23407 | Colon, NOS |
| TCGA-AA-3530 | TCGA-COAD | Adenocarcinoma, NOS | male | not reported | alive | -- | stage i | 29402 | Colon, NOS |
| TCGA-AA-3655 | TCGA-COAD | Adenocarcinoma, NOS | male | not reported | alive | -- | stage ii | 24896 | Sigmoid colon |
| TCGA-AA-3660 | TCGA-COAD | Adenocarcinoma, NOS | female | not reported | alive | -- | stage ii | 18932 | Colon, NOS |
| TCGA-AA-3662 | TCGA-COAD | Adenocarcinoma, NOS | female | not reported | alive | -- | stage iv | 29554 | Colon, NOS |
| TCGA-AA-3663 | TCGA-COAD | Adenocarcinoma, NOS | male | not reported | alive | -- | stage ii | 15675 | Cecum |
| TCGA-AA-3664 | TCGA-COAD | Adenocarcinoma, NOS | female | not reported | alive | -- | stage ii | 27363 | Ascending colon |
| TCGA-AA-3666 | TCGA-COAD | Adenocarcinoma, NOS | male | not reported | dead | 61 | stage iii | 25020 | Colon, NOS |
| TCGA-AA-3667 | TCGA-COAD | Adenocarcinoma, NOS | female | not reported | alive | -- | stage i | 13302 | Colon, NOS |
| TCGA-AA-3672 | TCGA-COAD | Adenocarcinoma, NOS | female | not reported | alive | -- | stage iii | 32872 | Transverse colon |
| TCGA-AA-3673 | TCGA-COAD | Adenocarcinoma, NOS | female | not reported | alive | -- | stage ii | 19509 | Transverse colon |
| TCGA-AA-3675 | TCGA-COAD | Adenocarcinoma, NOS | male | not reported | alive | -- | stage ii | 30801 | Ascending colon |
| TCGA-AA-3678 | TCGA-COAD | Adenocarcinoma, NOS | female | not reported | alive | -- | stage iii | 22219 | Sigmoid colon |
| TCGA-AA-3679 | TCGA-COAD | Adenocarcinoma, NOS | male | not reported | alive | -- | stage iv | 21611 | Colon, NOS |
| TCGA-AA-3680 | TCGA-COAD | Adenocarcinoma, NOS | female | not reported | dead | 335 | stage iv | 24655 | Cecum |
| TCGA-AA-3681 | TCGA-COAD | Adenocarcinoma, NOS | female | not reported | alive | -- | stage iii | 28399 | Cecum |
| TCGA-AA-3684 | TCGA-COAD | Mucinous adenocarcinoma | female | not reported | alive | -- | stage iv | 23741 | Cecum |
| TCGA-AA-3685 | TCGA-COAD | Adenocarcinoma, NOS | male | not reported | alive | -- | stage ii | 25506 | Colon, NOS |
| TCGA-AA-3688 | TCGA-COAD | Adenocarcinoma, NOS | male | not reported | alive | -- | stage iv | 29220 | Colon, NOS |
| TCGA-AA-3692 | TCGA-COAD | Mucinous adenocarcinoma | female | not reported | dead | 1095 | stage iv | 17167 | Colon, NOS |
| TCGA-AA-3693 | TCGA-COAD | Adenocarcinoma, NOS | female | not reported | alive | -- | stage iv | 28459 | Sigmoid colon |
| TCGA-AA-3695 | TCGA-COAD | Adenocarcinoma, NOS | female | not reported | alive | -- | stage iv | 23164 | Cecum |
| TCGA-AA-3696 | TCGA-COAD | Adenocarcinoma, NOS | female | not reported | dead | 153 | stage iv | 27667 | Sigmoid colon |
| TCGA-AA-3697 | TCGA-COAD | Adenocarcinoma, NOS | male | not reported | alive | -- | stage ii | 28367 | Colon, NOS |
| TCGA-AA-3712 | TCGA-COAD | Adenocarcinoma, NOS | male | not reported | alive | -- | stage iii | 23831 | Descending colon |
| TCGA-AA-3713 | TCGA-COAD | Adenocarcinoma, NOS | male | not reported | alive | -- | stage iv | 24927 | Colon, NOS |
| TCGA-AA-3715 | TCGA-COAD | Adenosquamous carcinoma | male | not reported | dead | 579 | stage ii | 28428 | Ascending colon |
| TCGA-AA-3811 | TCGA-COAD | Adenocarcinoma with neuroendocrine differentiation | female | not reported | dead | 306 | stage iii | 30893 | Colon, NOS |
| TCGA-AA-3812 | TCGA-COAD | Adenocarcinoma, NOS | female | not reported | alive | -- | stage iia | 29980 | Sigmoid colon |
| TCGA-AA-3814 | TCGA-COAD | Adenocarcinoma with mixed subtypes | female | not reported | alive | -- | stage iia | 31380 | Colon, NOS |
| TCGA-AA-3815 | TCGA-COAD | Adenocarcinoma, NOS | female | not reported | alive | -- | stage iia | 23922 | Cecum |
| TCGA-AA-3818 | TCGA-COAD | Adenocarcinoma, NOS | female | not reported | dead | 30 | stage iia | 28763 | Colon, NOS |
| TCGA-AA-3819 | TCGA-COAD | Adenocarcinoma, NOS | female | not reported | alive | -- | stage iia | 15280 | Colon, NOS |
| TCGA-AA-3821 | TCGA-COAD | Mucinous adenocarcinoma | female | not reported | alive | -- | stage i | 29706 | Ascending colon |
| TCGA-AA-3831 | TCGA-COAD | Adenocarcinoma, NOS | male | not reported | alive | -- | stage iia | 24411 | Colon, NOS |
| TCGA-AA-3833 | TCGA-COAD | Adenocarcinoma, NOS | female | not reported | alive | -- | stage iia | 23011 | Colon, NOS |
| TCGA-AA-3837 | TCGA-COAD | Mucinous adenocarcinoma | male | not reported | alive | -- | stage iia | 24655 | Colon, NOS |
| TCGA-AA-3841 | TCGA-COAD | Adenocarcinoma, NOS | male | not reported | alive | -- | stage iia | 24230 | Colon, NOS |
| TCGA-AA-3842 | TCGA-COAD | Adenocarcinoma, NOS | male | not reported | alive | -- | stage iiia | 18842 | Colon, NOS |
| TCGA-AA-3844 | TCGA-COAD | Adenocarcinoma, NOS | female | not reported | alive | -- | stage iiic | 28521 | Colon, NOS |
| TCGA-AA-3845 | TCGA-COAD | Adenocarcinoma, NOS | female | not reported | dead | 0 | stage iia | 31593 | Ascending colon |
| TCGA-AA-3846 | TCGA-COAD | Adenocarcinoma, NOS | female | not reported | alive | -- | stage iia | 27057 | Sigmoid colon |
| TCGA-AA-3848 | TCGA-COAD | Adenocarcinoma, NOS | female | not reported | dead | 306 | stage iiic | 30194 | Sigmoid colon |
| TCGA-AA-3850 | TCGA-COAD | Adenocarcinoma, NOS | male | not reported | dead | 0 | stage i | 27090 | Transverse colon |
| TCGA-AA-3851 | TCGA-COAD | Adenocarcinoma, NOS | male | not reported | alive | -- | stage iia | 27090 | Colon, NOS |
| TCGA-AA-3852 | TCGA-COAD | Mucinous adenocarcinoma | male | not reported | dead | 0 | stage iia | 32203 | Colon, NOS |
| TCGA-AA-3854 | TCGA-COAD | Mucinous adenocarcinoma | female | not reported | alive | -- | stage i | 26022 | Colon, NOS |
| TCGA-AA-3855 | TCGA-COAD | Adenocarcinoma, NOS | male | not reported | alive | -- | stage i | 26541 | Colon, NOS |
| TCGA-AA-3856 | TCGA-COAD | Adenocarcinoma, NOS | male | not reported | alive | -- | stage iia | 21670 | Colon, NOS |
| TCGA-AA-3858 | TCGA-COAD | Adenocarcinoma, NOS | male | not reported | alive | -- | stage i | 24806 | Colon, NOS |
| TCGA-AA-3860 | TCGA-COAD | Adenocarcinoma, NOS | female | not reported | alive | -- | stage iiib | 19449 | Sigmoid colon |
| TCGA-AA-3861 | TCGA-COAD | Adenocarcinoma, NOS | male | not reported | alive | -- | stage iia | 26602 | Cecum |
| TCGA-AA-3862 | TCGA-COAD | Adenocarcinoma, NOS | male | not reported | alive | -- | stage iia | 30163 | Ascending colon |
| TCGA-AA-3864 | TCGA-COAD | Adenocarcinoma, NOS | male | not reported | alive | -- | stage ii | 26237 | Cecum |
| TCGA-AA-3866 | TCGA-COAD | Adenocarcinoma, NOS | female | not reported | alive | -- | stage i | 28672 | Colon, NOS |
| TCGA-AA-3867 | TCGA-COAD | Adenocarcinoma, NOS | male | not reported | alive | -- | stage iv | 27028 | Sigmoid colon |
| TCGA-AA-3869 | TCGA-COAD | Adenocarcinoma, NOS | male | not reported | dead | 822 | stage iv | 28062 | Cecum |
| TCGA-AA-3870 | TCGA-COAD | Adenocarcinoma, NOS | female | not reported | alive | -- | stage iv | 26086 | Ascending colon |
| TCGA-AA-3872 | TCGA-COAD | Adenocarcinoma, NOS | male | not reported | alive | -- | stage iv | 16651 | Colon, NOS |
| TCGA-AA-3875 | TCGA-COAD | Adenocarcinoma, NOS | female | not reported | alive | -- | stage i | 28640 | Ascending colon |
| TCGA-AA-3877 | TCGA-COAD | Mucinous adenocarcinoma | female | not reported | alive | -- | stage i | 30377 | Transverse colon |
| TCGA-AA-3930 | TCGA-COAD | Adenocarcinoma, NOS | male | not reported | dead | 61 | stage iv | 24321 | Ascending colon |
| TCGA-AA-3939 | TCGA-COAD | Adenocarcinoma, NOS | male | not reported | alive | -- | stage iia | 30439 | Colon, NOS |
| TCGA-AA-3941 | TCGA-COAD | Adenocarcinoma, NOS | female | not reported | alive | -- | stage iva | 31015 | Colon, NOS |
| TCGA-AA-3947 | TCGA-COAD | Mucinous adenocarcinoma | female | not reported | alive | -- | stage iib | 22035 | Colon, NOS |
| TCGA-AA-3949 | TCGA-COAD | Mucinous adenocarcinoma | female | not reported | alive | -- | stage iiib | 32081 | Ascending colon |
| TCGA-AA-3950 | TCGA-COAD | Mucinous adenocarcinoma | female | not reported | alive | -- | stage iia | 29130 | Ascending colon |
| TCGA-AA-3952 | TCGA-COAD | Adenocarcinoma, NOS | male | not reported | dead | 61 | stage iiic | 24868 | Descending colon |
| TCGA-AA-3955 | TCGA-COAD | Adenocarcinoma, NOS | male | not reported | alive | -- | stage iiib | 14186 | Colon, NOS |
| TCGA-AA-3956 | TCGA-COAD | Adenocarcinoma, NOS | male | not reported | alive | -- | stage iia | 24045 | Cecum |
| TCGA-AA-3966 | TCGA-COAD | Mucinous adenocarcinoma | female | not reported | alive | -- | stage iia | 32537 | Colon, NOS |
| TCGA-AA-3967 | TCGA-COAD | Adenocarcinoma, NOS | male | not reported | alive | -- | stage iiib | 28216 | Colon, NOS |
| TCGA-AA-3968 | TCGA-COAD | Adenocarcinoma, NOS | female | not reported | alive | -- | stage i | 20089 | Sigmoid colon |
| TCGA-AA-3971 | TCGA-COAD | Adenocarcinoma, NOS | male | not reported | alive | -- | stage iii | 21518 | Colon, NOS |
| TCGA-AA-3972 | TCGA-COAD | Adenocarcinoma, NOS | male | not reported | alive | -- | stage iv | 26360 | Sigmoid colon |
| TCGA-AA-3973 | TCGA-COAD | Adenocarcinoma, NOS | male | not reported | alive | -- | stage iv | 25536 | Colon, NOS |
| TCGA-AA-3975 | TCGA-COAD | Adenocarcinoma, NOS | male | not reported | alive | -- | stage i | 29310 | Colon, NOS |
| TCGA-AA-3976 | TCGA-COAD | Adenocarcinoma, NOS | male | not reported | alive | -- | stage iiia | 25599 | Rectosigmoid junction |
| TCGA-AA-3977 | TCGA-COAD | Adenocarcinoma, NOS | male | not reported | alive | -- | not reported | 23832 | Sigmoid colon |
| TCGA-AA-3979 | TCGA-COAD | Adenocarcinoma, NOS | male | not reported | alive | -- | stage iia | 30742 | Sigmoid colon |
| TCGA-AA-3980 | TCGA-COAD | Adenocarcinoma, NOS | female | not reported | alive | -- | stage i | 32630 | Colon, NOS |
| TCGA-AA-3982 | TCGA-COAD | Adenocarcinoma, NOS | male | not reported | alive | -- | stage iiib | 27608 | Colon, NOS |
| TCGA-AA-3984 | TCGA-COAD | Adenocarcinoma, NOS | female | not reported | alive | -- | stage iia | 22585 | Sigmoid colon |
| TCGA-AA-3986 | TCGA-COAD | Adenocarcinoma, NOS | male | not reported | alive | -- | stage i | 26967 | Colon, NOS |
| TCGA-AA-3989 | TCGA-COAD | Adenocarcinoma, NOS | male | not reported | dead | 242 | stage iv | 30712 | Colon, NOS |
| TCGA-AA-3994 | TCGA-COAD | Mucinous adenocarcinoma | male | not reported | alive | -- | stage iiib | 25323 | Colon, NOS |
| TCGA-AA-A004 | TCGA-COAD | Adenocarcinoma, NOS | male | not reported | alive | -- | stage iia | 27943 | Sigmoid colon |
| TCGA-AA-A00N | TCGA-COAD | Mucinous adenocarcinoma | male | not reported | dead | 122 | stage iib | 27667 | Cecum |
| TCGA-AA-A010 | TCGA-COAD | Adenocarcinoma, NOS | female | not reported | alive | -- | stage iib | 16922 | Transverse colon |
| TCGA-AA-A017 | TCGA-COAD | Adenocarcinoma, NOS | female | not reported | alive | -- | stage iia | 21032 | Sigmoid colon |
| TCGA-AA-A01C | TCGA-COAD | Adenocarcinoma, NOS | male | not reported | alive | -- | stage iiia | 27729 | Ascending colon |
| TCGA-AA-A01I | TCGA-COAD | Adenocarcinoma, NOS | male | not reported | alive | -- | stage i | 27759 | Sigmoid colon |
| TCGA-AA-A01P | TCGA-COAD | Adenocarcinoma, NOS | female | not reported | dead | 1158 | stage iii | 29554 | Ascending colon |
| TCGA-AA-A01R | TCGA-COAD | Mucinous adenocarcinoma | male | not reported | alive | -- | stage iii | 17289 | Ascending colon |
| TCGA-AA-A01S | TCGA-COAD | Adenocarcinoma, NOS | female | not reported | alive | -- | stage iii | 17257 | Sigmoid colon |
| TCGA-AA-A01T | TCGA-COAD | Adenocarcinoma, NOS | female | not reported | alive | -- | stage iii | 23192 | Sigmoid colon |
| TCGA-AA-A01V | TCGA-COAD | Adenocarcinoma, NOS | male | not reported | alive | -- | stage i | 21792 | Cecum |
| TCGA-AA-A01X | TCGA-COAD | Adenocarcinoma, NOS | female | not reported | alive | -- | stage iii | 29281 | Sigmoid colon |
| TCGA-AA-A01Z | TCGA-COAD | Adenocarcinoma, NOS | male | not reported | alive | -- | stage ii | 24990 | Ascending colon |
| TCGA-AA-A022 | TCGA-COAD | Adenocarcinoma, NOS | female | not reported | alive | -- | stage ii | 32446 | Cecum |
| TCGA-AA-A024 | TCGA-COAD | Mucinous adenocarcinoma | male | not reported | dead | 1188 | stage ii | 29708 | Descending colon |
| TCGA-AA-A02E | TCGA-COAD | Adenocarcinoma, NOS | female | not reported | dead | 90 | stage iv | 30256 | Cecum |
| TCGA-AA-A02F | TCGA-COAD | Carcinoma, NOS | female | not reported | alive | -- | stage iv | 25051 | Sigmoid colon |
| TCGA-AA-A02H | TCGA-COAD | Adenocarcinoma, NOS | female | not reported | dead | 61 | stage iv | 27362 | Sigmoid colon |
| TCGA-AA-A02K | TCGA-COAD | Adenocarcinoma, NOS | male | not reported | dead | 426 | stage iv | 18506 | Ascending colon |
| TCGA-AA-A02O | TCGA-COAD | Adenocarcinoma, NOS | male | not reported | alive | -- | stage ii | 30288 | Transverse colon |
| TCGA-AA-A02R | TCGA-COAD | Adenocarcinoma, NOS | female | not reported | dead | 670 | stage iia | 30834 | Cecum |
| TCGA-AA-A02W | TCGA-COAD | Adenocarcinoma, NOS | female | not reported | alive | -- | stage i | 26693 | Sigmoid colon |
| TCGA-AA-A02Y | TCGA-COAD | Adenocarcinoma, NOS | male | not reported | alive | -- | stage i | 26724 | Cecum |
| TCGA-AA-A03F | TCGA-COAD | Mucinous adenocarcinoma | female | not reported | dead | 549 | stage iii | 32872 | Cecum |
| TCGA-AA-A03J | TCGA-COAD | Adenocarcinoma, NOS | female | not reported | alive | -- | stage i | 23986 | Sigmoid colon |
| TCGA-AD-5900 | TCGA-COAD | Mucinous adenocarcinoma | male | white | alive | -- | stage i | 24776 | Ascending colon |
| TCGA-AD-6548 | TCGA-COAD | Adenocarcinoma, NOS | female | white | alive | -- | stage i | 29758 | Splenic flexure of colon |
| TCGA-AD-6888 | TCGA-COAD | Adenocarcinoma, NOS | male | white | dead | 472 | stage iiib | 26816 | Ascending colon |
| TCGA-AD-6889 | TCGA-COAD | Adenocarcinoma, NOS | male | asian | dead | 2532 | stage iia | -- | Ascending colon |
| TCGA-AD-6890 | TCGA-COAD | Adenocarcinoma, NOS | male | white | alive | -- | not reported | 23769 | Ascending colon |
| TCGA-AD-6895 | TCGA-COAD | Adenocarcinoma, NOS | male | white | alive | -- | stage iiib | 30879 | Cecum |
| TCGA-AD-6899 | TCGA-COAD | Mucinous adenocarcinoma | male | white | dead | 176 | stage iiic | 30693 | Colon, NOS |
| TCGA-AD-6901 | TCGA-COAD | Adenocarcinoma, NOS | male | white | dead | 682 | not reported | 28579 | Cecum |
| TCGA-AD-6963 | TCGA-COAD | Adenocarcinoma, NOS | male | white | alive | -- | not reported | 21294 | Ascending colon |
| TCGA-AD-6964 | TCGA-COAD | Adenocarcinoma, NOS | male | white | dead | 331 | not reported | 21457 | Ascending colon |
| TCGA-AD-6965 | TCGA-COAD | Adenocarcinoma, NOS | male | white | alive | -- | stage iiic | 22853 | Cecum |
| TCGA-AD-A5EJ | TCGA-COAD | Adenocarcinoma, NOS | female | black or african american | alive | -- | stage iia | 27180 | Cecum |
| TCGA-AD-A5EK | TCGA-COAD | Adenocarcinoma, NOS | male | black or african american | alive | -- | stage i | 18731 | Ascending colon |
| TCGA-AF-2687 | TCGA-READ | Adenocarcinoma, NOS | male | white | alive | -- | stage iiic | 21098 | Rectosigmoid junction |
| TCGA-AF-2690 | TCGA-READ | Adenocarcinoma, NOS | female | white | dead | 524 | stage iiic | 28119 | Rectum, NOS |
| TCGA-AF-2693 | TCGA-READ | Adenocarcinoma, NOS | male | white | alive | -- | stage i | 27443 | Rectosigmoid junction |
| TCGA-AF-3911 | TCGA-READ | Adenocarcinoma, NOS | male | white | alive | -- | stage iiic | 17609 | Rectum, NOS |
| TCGA-AF-3913 | TCGA-READ | Adenocarcinoma, NOS | male | black or african american | dead | 316 | stage iv | 21970 | Rectosigmoid junction |
| TCGA-AF-3914 | TCGA-READ | Adenocarcinoma, NOS | male | white | alive | -- | stage iiib | 14426 | Rectosigmoid junction |
| TCGA-AF-4110 | TCGA-READ | Adenocarcinoma, NOS | male | white | alive | -- | stage iva | 28289 | Rectum, NOS |
| TCGA-AF-5654 | TCGA-READ | Adenocarcinoma, NOS | female | white | dead | 512 | stage i | 26738 | Rectosigmoid junction |
| TCGA-AF-6136 | TCGA-READ | Adenocarcinoma, NOS | female | white | alive | -- | stage iiib | 26490 | Rectosigmoid junction |
| TCGA-AF-6655 | TCGA-READ | Adenocarcinoma, NOS | male | white | alive | -- | stage iiia | 24417 | Rectosigmoid junction |
| TCGA-AF-6672 | TCGA-READ | Adenocarcinoma, NOS | male | white | alive | -- | stage iv | 15965 | Rectosigmoid junction |
| TCGA-AF-A56K | TCGA-READ | Adenocarcinoma, NOS | male | black or african american | alive | -- | stage iia | 20527 | Rectosigmoid junction |
| TCGA-AF-A56L | TCGA-READ | Adenocarcinoma, NOS | female | black or african american | alive | -- | stage iiic | 17734 | Rectosigmoid junction |
| TCGA-AF-A56N | TCGA-READ | Adenocarcinoma, NOS | female | black or african american | alive | -- | stage iia | 17480 | Rectosigmoid junction |
| TCGA-AG-3591 | TCGA-READ | Adenocarcinoma, NOS | female | not reported | alive | -- | stage iia | 24167 | Rectosigmoid junction |
| TCGA-AG-3592 | TCGA-READ | Adenocarcinoma, NOS | male | not reported | alive | -- | stage iia | 25141 | Rectosigmoid junction |
| TCGA-AG-3725 | TCGA-READ | Adenocarcinoma, NOS | female | not reported | alive | -- | stage iii | 32872 | Rectosigmoid junction |
| TCGA-AG-3726 | TCGA-READ | Adenocarcinoma, NOS | female | not reported | alive | -- | stage i | 23345 | Rectum, NOS |
| TCGA-AG-3727 | TCGA-READ | Adenocarcinoma, NOS | female | not reported | alive | -- | stage iii | 28641 | Rectosigmoid junction |
| TCGA-AG-3728 | TCGA-READ | Adenocarcinoma, NOS | male | not reported | alive | -- | stage iiib | 26906 | Rectum, NOS |
| TCGA-AG-3731 | TCGA-READ | Adenocarcinoma, NOS | male | not reported | alive | -- | stage iv | 23923 | Rectum, NOS |
| TCGA-AG-3732 | TCGA-READ | Adenocarcinoma, NOS | female | not reported | alive | -- | stage i | 28521 | Rectosigmoid junction |
| TCGA-AG-3742 | TCGA-READ | Adenocarcinoma, NOS | female | not reported | alive | -- | stage i | 31078 | Colon, NOS |
| TCGA-AG-3878 | TCGA-READ | Adenocarcinoma, NOS | male | not reported | alive | -- | stage i | 23527 | Rectum, NOS |
| TCGA-AG-3881 | TCGA-READ | Mucinous adenocarcinoma | female | not reported | alive | -- | stage iia | 30467 | Unknown primary site |
| TCGA-AG-3882 | TCGA-READ | Adenocarcinoma, NOS | female | not reported | alive | -- | stage i | 24198 | Colon, NOS |
| TCGA-AG-3883 | TCGA-READ | Adenocarcinoma, NOS | male | not reported | alive | -- | stage i | 25415 | Rectum, NOS |
| TCGA-AG-3885 | TCGA-READ | Adenocarcinoma, NOS | female | not reported | alive | -- | stage iiib | 26145 | Rectum, NOS |
| TCGA-AG-3887 | TCGA-READ | Mucinous adenocarcinoma | male | not reported | alive | -- | stage iia | 24960 | Rectum, NOS |
| TCGA-AG-3890 | TCGA-READ | Adenocarcinoma, NOS | male | not reported | alive | -- | stage i | 22950 | Rectum, NOS |
| TCGA-AG-3891 | TCGA-READ | Adenocarcinoma, NOS | female | not reported | alive | -- | stage i | 24411 | Rectum, NOS |
| TCGA-AG-3892 | TCGA-READ | Adenocarcinoma, NOS | female | not reported | alive | -- | stage i | 20880 | Rectum, NOS |
| TCGA-AG-3893 | TCGA-READ | Adenocarcinoma, NOS | male | not reported | alive | -- | stage iiib | 27272 | Rectosigmoid junction |
| TCGA-AG-3894 | TCGA-READ | Adenocarcinoma, NOS | male | not reported | alive | -- | stage iia | 23954 | Rectum, NOS |
| TCGA-AG-3896 | TCGA-READ | Adenocarcinoma, NOS | female | not reported | alive | -- | stage i | 31046 | Rectum, NOS |
| TCGA-AG-3898 | TCGA-READ | Adenocarcinoma, NOS | male | not reported | alive | -- | stage iia | 22492 | Rectum, NOS |
| TCGA-AG-3901 | TCGA-READ | Mucinous adenocarcinoma | female | not reported | alive | -- | stage iiib | 24502 | Rectosigmoid junction |
| TCGA-AG-3902 | TCGA-READ | Adenocarcinoma, NOS | male | not reported | alive | -- | stage iia | 22492 | Rectosigmoid junction |
| TCGA-AG-3906 | TCGA-READ | Adenocarcinoma, NOS | female | not reported | alive | -- | stage i | 24806 | Rectosigmoid junction |
| TCGA-AG-3909 | TCGA-READ | Adenocarcinoma, NOS | female | not reported | alive | -- | stage iiib | 25537 | Rectosigmoid junction |
| TCGA-AG-4001 | TCGA-READ | Adenocarcinoma, NOS | female | not reported | alive | -- | stage iia | 27302 | Rectosigmoid junction |
| TCGA-AG-4008 | TCGA-READ | Adenocarcinoma, NOS | male | not reported | alive | -- | stage iia | 23192 | Colon, NOS |
| TCGA-AG-4009 | TCGA-READ | Adenocarcinoma, NOS | male | not reported | alive | -- | stage i | 30559 | Rectum, NOS |
| TCGA-AG-4015 | TCGA-READ | Adenocarcinoma, NOS | female | not reported | alive | -- | stage iia | 31291 | Colon, NOS |
| TCGA-AG-4021 | TCGA-READ | Adenocarcinoma, NOS | female | not reported | dead | 121 | stage iv | 31015 | Rectum, NOS |
| TCGA-AG-4022 | TCGA-READ | Adenocarcinoma, NOS | female | not reported | alive | -- | stage ii | 21823 | Rectum, NOS |
| TCGA-AG-A002 | TCGA-READ | Adenocarcinoma, NOS | male | not reported | alive | -- | stage i | 13090 | Rectum, NOS |
| TCGA-AG-A008 | TCGA-READ | Mucinous adenocarcinoma | female | not reported | alive | -- | stage i | 18416 | Rectum, NOS |
| TCGA-AG-A00C | TCGA-READ | Adenocarcinoma, NOS | female | not reported | alive | -- | stage iiib | 18201 | Rectum, NOS |
| TCGA-AG-A00Y | TCGA-READ | Adenocarcinoma, NOS | male | not reported | alive | -- | stage iia | 24837 | Rectum, NOS |
| TCGA-AG-A011 | TCGA-READ | Adenocarcinoma, NOS | male | not reported | alive | -- | stage iia | 29463 | Rectum, NOS |
| TCGA-AG-A014 | TCGA-READ | Adenocarcinoma, NOS | male | not reported | alive | -- | stage i | 31718 | Rectum, NOS |
| TCGA-AG-A015 | TCGA-READ | Adenocarcinoma, NOS | female | not reported | alive | -- | stage i | 23496 | Rectum, NOS |
| TCGA-AG-A016 | TCGA-READ | Adenocarcinoma, NOS | male | not reported | alive | -- | stage iv | 20178 | Rectum, NOS |
| TCGA-AG-A01J | TCGA-READ | Adenocarcinoma, NOS | female | not reported | alive | -- | stage iia | 21581 | Rectum, NOS |
| TCGA-AG-A01L | TCGA-READ | Adenocarcinoma, NOS | male | not reported | alive | -- | stage iiib | 21369 | Rectum, NOS |
| TCGA-AG-A01N | TCGA-READ | Adenocarcinoma, NOS | female | not reported | alive | -- | stage iv | 25049 | Rectum, NOS |
| TCGA-AG-A01W | TCGA-READ | Adenocarcinoma, NOS | female | not reported | alive | -- | stage ii | 24745 | Rectum, NOS |
| TCGA-AG-A01Y | TCGA-READ | Adenocarcinoma, NOS | female | not reported | alive | -- | stage ii | 18112 | Rectum, NOS |
| TCGA-AG-A020 | TCGA-READ | Mucinous adenocarcinoma | female | not reported | alive | -- | stage iii | 20851 | Rectum, NOS |
| TCGA-AG-A023 | TCGA-READ | Adenocarcinoma, NOS | female | not reported | dead | 1581 | stage iv | 22830 | Rectum, NOS |
| TCGA-AG-A026 | TCGA-READ | Adenocarcinoma, NOS | male | not reported | dead | 59 | stage ii | 24413 | Rectum, NOS |
| TCGA-AG-A02N | TCGA-READ | Adenocarcinoma, NOS | male | not reported | alive | -- | stage ii | 24687 | Rectum, NOS |
| TCGA-AG-A02X | TCGA-READ | Adenocarcinoma, NOS | male | not reported | alive | -- | stage i | 28399 | Rectum, NOS |
| TCGA-AG-A032 | TCGA-READ | Adenocarcinoma, NOS | male | not reported | alive | -- | stage iiib | 24865 | Rectum, NOS |
| TCGA-AG-A036 | TCGA-READ | Adenocarcinoma, NOS | male | not reported | alive | -- | stage iii | 26206 | Rectum, NOS |
| TCGA-AH-6544 | TCGA-READ | Adenocarcinoma, NOS | male | white | alive | -- | not reported | 21977 | Rectosigmoid junction |
| TCGA-AH-6547 | TCGA-READ | Adenocarcinoma, NOS | female | white | dead | 76 | not reported | 29030 | Rectosigmoid junction |
| TCGA-AH-6549 | TCGA-READ | Adenocarcinoma, NOS | male | white | alive | -- | not reported | 24337 | Rectum, NOS |
| TCGA-AH-6643 | TCGA-READ | Adenocarcinoma, NOS | male | white | dead | 1314 | stage iiic | 18273 | Rectosigmoid junction |
| TCGA-AH-6644 | TCGA-READ | Adenocarcinoma, NOS | male | white | alive | -- | not reported | 26892 | Rectosigmoid junction |
| TCGA-AH-6897 | TCGA-READ | Adenocarcinoma, NOS | male | white | alive | -- | stage i | 17536 | Rectosigmoid junction |
| TCGA-AH-6903 | TCGA-READ | Mucinous adenocarcinoma | male | white | alive | -- | stage iiib | 17030 | Rectosigmoid junction |
| TCGA-AM-5820 | TCGA-COAD | Adenocarcinoma, NOS | female | white | alive | -- | stage iva | 21902 | Colon, NOS |
| TCGA-AM-5821 | TCGA-COAD | Adenocarcinoma, NOS | female | white | alive | -- | stage iia | 24903 | Sigmoid colon |
| TCGA-AU-3779 | TCGA-COAD | Adenocarcinoma, NOS | female | white | alive | -- | stage iia | 29460 | Sigmoid colon |
| TCGA-AU-6004 | TCGA-COAD | Adenocarcinoma, NOS | female | white | alive | -- | stage i | 25259 | Cecum |
| TCGA-AY-4070 | TCGA-COAD | Adenocarcinoma, NOS | female | black or african american | dead | 496 | stage iiic | 18621 | Cecum |
| TCGA-AY-4071 | TCGA-COAD | Adenocarcinoma, NOS | female | white | dead | 29 | stage i | 23320 | Sigmoid colon |
| TCGA-AY-5543 | TCGA-COAD | Adenocarcinoma, NOS | female | black or african american | alive | -- | stage iva | 23870 | Ascending colon |
| TCGA-AY-6196 | TCGA-COAD | Mucinous adenocarcinoma | male | white | alive | -- | stage iiic | 17311 | Ascending colon |
| TCGA-AY-6197 | TCGA-COAD | Adenocarcinoma, NOS | male | white | alive | -- | stage iia | 21957 | Ascending colon |
| TCGA-AY-6386 | TCGA-COAD | Adenocarcinoma, NOS | female | black or african american | alive | -- | stage iiib | 24453 | Cecum |
| TCGA-AY-A54L | TCGA-COAD | Adenocarcinoma, NOS | female | black or african american | alive | -- | stage i | 27074 | Hepatic flexure of colon |
| TCGA-AY-A69D | TCGA-COAD | Adenocarcinoma, NOS | female | black or african american | alive | -- | stage iia | 20299 | Transverse colon |
| TCGA-AY-A71X | TCGA-COAD | Adenocarcinoma, NOS | female | black or african american | alive | -- | stage i | 19904 | Cecum |
| TCGA-AY-A8YK | TCGA-COAD | Adenocarcinoma, NOS | male | black or african american | alive | -- | stage iva | 16140 | Sigmoid colon |
| TCGA-AZ-4308 | TCGA-COAD | Adenocarcinoma, NOS | female | white | alive | -- | stage iiib | 17321 | Sigmoid colon |
| TCGA-AZ-4313 | TCGA-COAD | Adenocarcinoma, NOS | female | white | alive | -- | stage i | 18765 | Descending colon |
| TCGA-AZ-4315 | TCGA-COAD | Adenocarcinoma, NOS | male | white | alive | -- | stage iia | 22340 | Cecum |
| TCGA-AZ-4323 | TCGA-COAD | Adenocarcinoma, NOS | male | white | dead | 43 | stage iv | 13755 | Cecum |
| TCGA-AZ-4614 | TCGA-COAD | Adenocarcinoma, NOS | female | white | dead | 172 | stage iva | 26267 | Cecum |
| TCGA-AZ-4615 | TCGA-COAD | Adenocarcinoma, NOS | male | white | alive | -- | stage iiib | 30908 | Cecum |
| TCGA-AZ-4616 | TCGA-COAD | Adenocarcinoma, NOS | female | white | dead | 156 | stage iv | 30043 | Cecum |
| TCGA-AZ-4681 | TCGA-COAD | Adenocarcinoma, NOS | female | white | alive | -- | stage iia | 28906 | Ascending colon |
| TCGA-AZ-4682 | TCGA-COAD | Adenocarcinoma, NOS | male | white | dead | 680 | stage iva | 22409 | Rectosigmoid junction |
| TCGA-AZ-4684 | TCGA-COAD | Adenocarcinoma, NOS | male | white | alive | -- | stage iva | 18252 | Rectosigmoid junction |
| TCGA-AZ-5403 | TCGA-COAD | Adenocarcinoma, NOS | male | white | dead | 1910 | stage ii | 15983 | Descending colon |
| TCGA-AZ-5407 | TCGA-COAD | Adenocarcinoma, NOS | female | white | alive | -- | stage i | 18820 | Cecum |
| TCGA-AZ-6598 | TCGA-COAD | Adenocarcinoma, NOS | female | white | dead | 1503 | stage ii | 28182 | Ascending colon |
| TCGA-AZ-6599 | TCGA-COAD | Adenocarcinoma, NOS | male | white | dead | 206 | stage i | 26438 | Cecum |
| TCGA-AZ-6600 | TCGA-COAD | Adenocarcinoma, NOS | male | black or african american | dead | 368 | stage iv | 23581 | Ascending colon |
| TCGA-AZ-6601 | TCGA-COAD | Adenocarcinoma, NOS | male | white | dead | 3042 | stage ii | 25082 | Sigmoid colon |
| TCGA-AZ-6603 | TCGA-COAD | Adenocarcinoma, NOS | female | white | dead | 899 | not reported | 28424 | Sigmoid colon |
| TCGA-AZ-6605 | TCGA-COAD | Adenocarcinoma, NOS | male | white | dead | 159 | stage iiib | 28485 | Ascending colon |
| TCGA-AZ-6606 | TCGA-COAD | Adenocarcinoma, NOS | male | white | dead | 357 | stage iv | 29704 | Cecum |
| TCGA-AZ-6607 | TCGA-COAD | Adenocarcinoma, NOS | male | white | dead | 97 | stage iv | 25448 | Sigmoid colon |
| TCGA-AZ-6608 | TCGA-COAD | Adenocarcinoma, NOS | female | white | dead | 59 | stage iiia | 20094 | Sigmoid colon |
| TCGA-BM-6198 | TCGA-READ | Adenocarcinoma, NOS | male | white | alive | -- | stage iiib | 26837 | Rectum, NOS |
| TCGA-CA-5254 | TCGA-COAD | Adenocarcinoma, NOS | female | asian | alive | -- | stage iia | 15415 | Colon, NOS |
| TCGA-CA-5255 | TCGA-COAD | Adenocarcinoma, NOS | male | asian | alive | -- | stage iia | 16743 | Colon, NOS |
| TCGA-CA-5256 | TCGA-COAD | Adenocarcinoma, NOS | female | asian | alive | -- | stage iia | 19785 | Colon, NOS |
| TCGA-CA-5796 | TCGA-COAD | Mucinous adenocarcinoma | female | asian | alive | -- | stage iia | 19353 | Ascending colon |
| TCGA-CA-5797 | TCGA-COAD | Adenocarcinoma, NOS | male | asian | alive | -- | stage iia | 20662 | Colon, NOS |
| TCGA-CA-6715 | TCGA-COAD | Papillary adenocarcinoma, NOS | male | asian | alive | -- | stage iiib | 23075 | Sigmoid colon |
| TCGA-CA-6716 | TCGA-COAD | Papillary adenocarcinoma, NOS | male | asian | alive | -- | stage iia | 23943 | Ascending colon |
| TCGA-CA-6717 | TCGA-COAD | Mucinous adenocarcinoma | male | asian | alive | -- | stage iia | 20984 | Ascending colon |
| TCGA-CA-6718 | TCGA-COAD | Adenocarcinoma, NOS | male | asian | dead | 306 | stage iia | 17109 | Colon, NOS |
| TCGA-CA-6719 | TCGA-COAD | Adenocarcinoma, NOS | male | asian | alive | -- | stage iia | 28343 | Descending colon |
| TCGA-CI-6619 | TCGA-READ | Adenocarcinoma, NOS | male | white | alive | -- | stage iv | 15322 | Rectum, NOS |
| TCGA-CI-6620 | TCGA-READ | Adenocarcinoma, NOS | female | white | alive | -- | stage iva | 15322 | Rectum, NOS |
| TCGA-CI-6621 | TCGA-READ | Adenocarcinoma, NOS | male | white | alive | -- | stage iiib | 23240 | Rectum, NOS |
| TCGA-CI-6622 | TCGA-READ | Adenocarcinoma, NOS | male | white | alive | -- | stage iib | 27336 | Rectum, NOS |
| TCGA-CI-6623 | TCGA-READ | Adenocarcinoma, NOS | male | white | alive | -- | stage i | 16177 | Rectum, NOS |
| TCGA-CI-6624 | TCGA-READ | Adenocarcinoma, NOS | female | white | alive | -- | stage i | 19419 | Rectum, NOS |
| TCGA-CK-4947 | TCGA-COAD | Adenocarcinoma, NOS | female | white | alive | -- | stage iiib | 16980 | Sigmoid colon |
| TCGA-CK-4948 | TCGA-COAD | Adenocarcinoma, NOS | female | white | alive | -- | stage iii | 16736 | Sigmoid colon |
| TCGA-CK-4950 | TCGA-COAD | Mucinous adenocarcinoma | female | black or african american | alive | -- | stage iiib | 24944 | Cecum |
| TCGA-CK-4951 | TCGA-COAD | Mucinous adenocarcinoma | female | white | dead | 2134 | stage iia | 28982 | Cecum |
| TCGA-CK-4952 | TCGA-COAD | Mucinous adenocarcinoma | female | white | alive | -- | stage iiic | 17826 | Ascending colon |
| TCGA-CK-5912 | TCGA-COAD | Adenocarcinoma, NOS | male | white | dead | 1493 | stage i | 29938 | Ascending colon |
| TCGA-CK-5913 | TCGA-COAD | Adenocarcinoma, NOS | female | white | alive | -- | stage iia | 21399 | Ascending colon |
| TCGA-CK-5914 | TCGA-COAD | Adenocarcinoma, NOS | male | white | alive | -- | stage iiib | 29586 | Sigmoid colon |
| TCGA-CK-5915 | TCGA-COAD | Adenocarcinoma, NOS | male | white | alive | -- | stage i | 23040 | Sigmoid colon |
| TCGA-CK-5916 | TCGA-COAD | Adenocarcinoma, NOS | female | white | dead | 643 | stage i | 26024 | Ascending colon |
| TCGA-CK-6746 | TCGA-COAD | Adenocarcinoma, NOS | female | white | alive | -- | stage iib | 30957 | Cecum |
| TCGA-CK-6747 | TCGA-COAD | Adenocarcinoma, NOS | female | white | alive | -- | stage iia | 32052 | Cecum |
| TCGA-CK-6748 | TCGA-COAD | Mucinous adenocarcinoma | female | white | alive | -- | stage iv | 16529 | Sigmoid colon |
| TCGA-CK-6751 | TCGA-COAD | Mucinous adenocarcinoma | female | white | alive | -- | stage i | 32354 | Ascending colon |
| TCGA-CL-4957 | TCGA-READ | Adenocarcinoma, NOS | female | not reported | dead | -- | not reported | 29081 | Rectum, NOS |
| TCGA-CL-5917 | TCGA-READ | Adenocarcinoma, NOS | female | white | alive | -- | stage iiic | 26084 | Rectosigmoid junction |
| TCGA-CL-5918 | TCGA-READ | Adenocarcinoma, NOS | female | white | alive | -- | stage iia | 32872 | Rectum, NOS |
| TCGA-CM-4743 | TCGA-COAD | Adenocarcinoma, NOS | male | white | alive | -- | stage iia | 25291 | Ascending colon |
| TCGA-CM-4744 | TCGA-COAD | Adenocarcinoma, NOS | male | white | alive | -- | stage i | 25506 | Cecum |
| TCGA-CM-4746 | TCGA-COAD | Adenocarcinoma, NOS | male | black or african american | alive | -- | stage i | 22403 | Sigmoid colon |
| TCGA-CM-4747 | TCGA-COAD | Adenocarcinoma, NOS | male | white | alive | -- | stage iva | 17198 | Cecum |
| TCGA-CM-4748 | TCGA-COAD | Mucinous adenocarcinoma | male | white | alive | -- | stage iiib | 19509 | Transverse colon |
| TCGA-CM-4750 | TCGA-COAD | Adenocarcinoma, NOS | female | white | alive | -- | stage iiia | 12478 | Rectosigmoid junction |
| TCGA-CM-4751 | TCGA-COAD | Adenocarcinoma, NOS | male | white | alive | -- | stage iiib | 22891 | Cecum |
| TCGA-CM-4752 | TCGA-COAD | Adenocarcinoma, NOS | male | black or african american | alive | -- | stage iia | 21519 | Ascending colon |
| TCGA-CM-5341 | TCGA-COAD | Adenocarcinoma, NOS | female | white | alive | -- | stage iiia | 30163 | Sigmoid colon |
| TCGA-CM-5344 | TCGA-COAD | Adenocarcinoma, NOS | female | white | alive | -- | stage iiib | 14426 | Sigmoid colon |
| TCGA-CM-5348 | TCGA-COAD | Adenocarcinoma, NOS | male | white | alive | -- | stage iiib | 26543 | Cecum |
| TCGA-CM-5349 | TCGA-COAD | Adenocarcinoma, NOS | female | white | alive | -- | stage iia | 24867 | Cecum |
| TCGA-CM-5860 | TCGA-COAD | Adenocarcinoma, NOS | male | white | alive | -- | stage iia | 16344 | Ascending colon |
| TCGA-CM-5861 | TCGA-COAD | Adenocarcinoma, NOS | female | white | alive | -- | stage iia | 23253 | Cecum |
| TCGA-CM-5862 | TCGA-COAD | Adenocarcinoma, NOS | male | white | dead | 153 | stage iva | 29493 | Ascending colon |
| TCGA-CM-5863 | TCGA-COAD | Mucinous adenocarcinoma | female | white | alive | -- | stage iiib | 22127 | Ascending colon |
| TCGA-CM-5864 | TCGA-COAD | Adenocarcinoma, NOS | male | white | alive | -- | stage i | 22219 | Cecum |
| TCGA-CM-5868 | TCGA-COAD | Adenocarcinoma, NOS | female | white | alive | -- | stage iva | 21672 | Sigmoid colon |
| TCGA-CM-6161 | TCGA-COAD | Adenocarcinoma, NOS | female | white | alive | -- | stage i | 13363 | Sigmoid colon |
| TCGA-CM-6162 | TCGA-COAD | Mucinous adenocarcinoma | female | white | alive | -- | stage iiib | 17624 | Ascending colon |
| TCGA-CM-6163 | TCGA-COAD | Adenocarcinoma, NOS | male | white | alive | -- | stage i | 27150 | Sigmoid colon |
| TCGA-CM-6164 | TCGA-COAD | Adenocarcinoma, NOS | female | white | alive | -- | stage iia | 16802 | Sigmoid colon |
| TCGA-CM-6165 | TCGA-COAD | Adenocarcinoma, NOS | male | white | alive | -- | stage iia | 27302 | Sigmoid colon |
| TCGA-CM-6166 | TCGA-COAD | Adenocarcinoma, NOS | female | white | alive | -- | stage i | 17807 | Ascending colon |
| TCGA-CM-6167 | TCGA-COAD | Adenocarcinoma, NOS | female | black or african american | alive | -- | stage iiic | 21031 | Cecum |
| TCGA-CM-6168 | TCGA-COAD | Adenocarcinoma, NOS | female | black or african american | alive | -- | stage iia | 30834 | Ascending colon |
| TCGA-CM-6169 | TCGA-COAD | Adenocarcinoma, NOS | male | black or african american | alive | -- | stage iia | 24564 | Cecum |
| TCGA-CM-6170 | TCGA-COAD | Adenocarcinoma, NOS | female | white | alive | -- | stage i | 26663 | Descending colon |
| TCGA-CM-6171 | TCGA-COAD | Adenocarcinoma, NOS | female | white | alive | -- | stage i | 28307 | Ascending colon |
| TCGA-CM-6172 | TCGA-COAD | Adenocarcinoma, NOS | female | white | alive | -- | stage iiib | 25902 | Sigmoid colon |
| TCGA-CM-6674 | TCGA-COAD | Adenocarcinoma, NOS | male | white | alive | -- | stage iia | 14368 | Hepatic flexure of colon |
| TCGA-CM-6675 | TCGA-COAD | Adenocarcinoma, NOS | male | black or african american | alive | -- | stage ivb | 12935 | Cecum |
| TCGA-CM-6676 | TCGA-COAD | Adenocarcinoma, NOS | male | white | alive | -- | stage i | 30223 | Sigmoid colon |
| TCGA-CM-6677 | TCGA-COAD | Adenocarcinoma, NOS | female | white | alive | -- | stage iia | 27575 | Hepatic flexure of colon |
| TCGA-CM-6678 | TCGA-COAD | Adenocarcinoma, NOS | female | white | alive | -- | stage iva | 23070 | Sigmoid colon |
| TCGA-CM-6679 | TCGA-COAD | Adenocarcinoma, NOS | male | white | alive | -- | stage iia | 21519 | Sigmoid colon |
| TCGA-CM-6680 | TCGA-COAD | Adenocarcinoma, NOS | female | white | alive | -- | stage iiib | 28701 | Cecum |
| TCGA-D5-5537 | TCGA-COAD | Adenocarcinoma, NOS | male | white | dead | 1381 | not reported | 30505 | Colon, NOS |
| TCGA-D5-5538 | TCGA-COAD | Adenocarcinoma, NOS | female | white | dead | 1661 | stage iiib | 21974 | Cecum |
| TCGA-D5-5539 | TCGA-COAD | Mucinous adenocarcinoma | male | white | alive | -- | stage iiia | 22097 | Ascending colon |
| TCGA-D5-5540 | TCGA-COAD | Adenocarcinoma, NOS | male | white | alive | -- | stage iia | 26972 | Cecum |
| TCGA-D5-5541 | TCGA-COAD | Adenocarcinoma, NOS | male | white | alive | -- | stage iiib | 23109 | Sigmoid colon |
| TCGA-D5-6529 | TCGA-COAD | Adenocarcinoma, NOS | male | white | alive | -- | stage iia | 25394 | Colon, NOS |
| TCGA-D5-6530 | TCGA-COAD | Adenocarcinoma, NOS | male | white | alive | -- | stage i | 19591 | Cecum |
| TCGA-D5-6531 | TCGA-COAD | Adenocarcinoma, NOS | male | white | alive | -- | stage iia | 27633 | Hepatic flexure of colon |
| TCGA-D5-6532 | TCGA-COAD | Adenocarcinoma, NOS | male | white | alive | -- | stage iia | 22344 | Sigmoid colon |
| TCGA-D5-6533 | TCGA-COAD | Adenocarcinoma, NOS | female | white | alive | -- | not reported | 24971 | Transverse colon |
| TCGA-D5-6534 | TCGA-COAD | Mucinous adenocarcinoma | female | white | alive | -- | stage iia | 22778 | Colon, NOS |
| TCGA-D5-6535 | TCGA-COAD | Adenocarcinoma, NOS | female | white | alive | -- | stage iiib | 29278 | Colon, NOS |
| TCGA-D5-6536 | TCGA-COAD | Adenocarcinoma, NOS | male | white | alive | -- | stage iia | 26890 | Colon, NOS |
| TCGA-D5-6537 | TCGA-COAD | Adenocarcinoma, NOS | male | white | dead | 146 | stage iiib | 23381 | Transverse colon |
| TCGA-D5-6538 | TCGA-COAD | Adenocarcinoma, NOS | female | white | alive | -- | stage iiib | 29194 | Hepatic flexure of colon |
| TCGA-D5-6539 | TCGA-COAD | Adenocarcinoma, NOS | female | white | alive | -- | not reported | 16703 | Transverse colon |
| TCGA-D5-6540 | TCGA-COAD | Mucinous adenocarcinoma | male | white | alive | -- | stage i | 24282 | Cecum |
| TCGA-D5-6541 | TCGA-COAD | Adenocarcinoma, NOS | male | white | alive | -- | stage iia | 18029 | Splenic flexure of colon |
| TCGA-D5-6898 | TCGA-COAD | Adenocarcinoma, NOS | female | white | alive | -- | stage i | 18874 | Colon, NOS |
| TCGA-D5-6920 | TCGA-COAD | Adenocarcinoma, NOS | female | white | alive | -- | stage iia | 28124 | Sigmoid colon |
| TCGA-D5-6922 | TCGA-COAD | Adenocarcinoma, NOS | male | white | alive | -- | stage iiia | 27771 | Sigmoid colon |
| TCGA-D5-6924 | TCGA-COAD | Adenocarcinoma, NOS | male | white | alive | -- | stage iia | 24964 | Sigmoid colon |
| TCGA-D5-6926 | TCGA-COAD | Adenocarcinoma, NOS | male | white | alive | -- | stage iiib | 23768 | Sigmoid colon |
| TCGA-D5-6927 | TCGA-COAD | Adenocarcinoma, NOS | male | white | alive | -- | stage iia | 12737 | Sigmoid colon |
| TCGA-D5-6928 | TCGA-COAD | Mucinous adenocarcinoma | male | white | alive | -- | stage iia | 29411 | Hepatic flexure of colon |
| TCGA-D5-6929 | TCGA-COAD | Adenocarcinoma, NOS | female | white | alive | -- | stage iv | 17919 | Colon, NOS |
| TCGA-D5-6930 | TCGA-COAD | Mucinous adenocarcinoma | male | white | alive | -- | stage iia | 24764 | Cecum |
| TCGA-D5-6931 | TCGA-COAD | Adenocarcinoma, NOS | male | white | alive | -- | stage iiic | 28292 | Colon, NOS |
| TCGA-D5-6932 | TCGA-COAD | Adenocarcinoma, NOS | male | white | alive | -- | stage iia | 25466 | Colon, NOS |
| TCGA-D5-7000 | TCGA-COAD | Mucinous adenocarcinoma | female | white | alive | -- | stage i | 28913 | Cecum |
| TCGA-DC-4745 | TCGA-READ | Adenocarcinoma, NOS | female | white | alive | -- | stage iiib | 17958 | Rectosigmoid junction |
| TCGA-DC-4749 | TCGA-READ | Adenocarcinoma, NOS | male | white | alive | -- | stage i | 21092 | Rectosigmoid junction |
| TCGA-DC-5337 | TCGA-READ | Adenocarcinoma, NOS | male | white | alive | -- | stage i | 25202 | Rectosigmoid junction |
| TCGA-DC-5869 | TCGA-READ | Adenocarcinoma, NOS | female | black or african american | alive | -- | stage iiib | 22676 | Rectum, NOS |
| TCGA-DC-6154 | TCGA-READ | Adenocarcinoma, NOS | female | white | alive | -- | stage iva | 21093 | Rectosigmoid junction |
| TCGA-DC-6155 | TCGA-READ | Adenocarcinoma, NOS | female | white | alive | -- | stage iiia | 11381 | Rectosigmoid junction |
| TCGA-DC-6157 | TCGA-READ | Adenocarcinoma, NOS | male | black or african american | alive | -- | stage i | 17807 | Rectosigmoid junction |
| TCGA-DC-6158 | TCGA-READ | Adenocarcinoma, NOS | male | white | dead | 334 | stage i | 25842 | Rectum, NOS |
| TCGA-DC-6160 | TCGA-READ | Adenocarcinoma, NOS | male | white | alive | -- | stage i | 24990 | Rectosigmoid junction |
| TCGA-DC-6681 | TCGA-READ | Adenocarcinoma, NOS | female | white | alive | -- | stage iva | 25659 | Rectosigmoid junction |
| TCGA-DC-6682 | TCGA-READ | Adenocarcinoma, NOS | male | white | alive | -- | stage iia | 21062 | Rectosigmoid junction |
| TCGA-DC-6683 | TCGA-READ | Adenocarcinoma, NOS | male | white | alive | -- | stage iiib | 15918 | Rectosigmoid junction |
| TCGA-DM-A0X9 | TCGA-COAD | Adenocarcinoma, NOS | female | white | alive | -- | stage iia | 26217 | Ascending colon |
| TCGA-DM-A0XD | TCGA-COAD | Adenocarcinoma, NOS | male | white | dead | 743 | stage iia | 23966 | Cecum |
| TCGA-DM-A0XF | TCGA-COAD | Adenocarcinoma, NOS | female | white | dead | 1162 | stage iiic | 25032 | Sigmoid colon |
| TCGA-DM-A1D0 | TCGA-COAD | Adenocarcinoma, NOS | female | white | alive | -- | stage iia | 28875 | Sigmoid colon |
| TCGA-DM-A1D4 | TCGA-COAD | Adenocarcinoma, NOS | male | white | dead | 2821 | stage iia | 29403 | Cecum |
| TCGA-DM-A1D6 | TCGA-COAD | Mucinous adenocarcinoma | male | white | dead | 1518 | stage iia | 32302 | Splenic flexure of colon |
| TCGA-DM-A1D7 | TCGA-COAD | Mucinous adenocarcinoma | male | white | dead | 405 | stage iia | 30142 | Sigmoid colon |
| TCGA-DM-A1D8 | TCGA-COAD | Adenocarcinoma, NOS | female | white | dead | 383 | not reported | 18311 | Ascending colon |
| TCGA-DM-A1D9 | TCGA-COAD | Adenocarcinoma, NOS | female | white | alive | -- | stage iia | 24514 | Cecum |
| TCGA-DM-A1DA | TCGA-COAD | Adenocarcinoma, NOS | female | white | dead | 228 | stage iiic | 26169 | Cecum |
| TCGA-DM-A1DB | TCGA-COAD | Adenocarcinoma, NOS | male | white | dead | 1348 | stage iia | 24888 | Sigmoid colon |
| TCGA-DM-A1HA | TCGA-COAD | Adenocarcinoma, NOS | male | white | alive | -- | stage iiic | 30272 | Ascending colon |
| TCGA-DM-A1HB | TCGA-COAD | Mucinous adenocarcinoma | male | white | alive | -- | stage iiib | 27708 | Transverse colon |
| TCGA-DM-A280 | TCGA-COAD | Mucinous adenocarcinoma | female | white | dead | 236 | stage iia | 25649 | Ascending colon |
| TCGA-DM-A282 | TCGA-COAD | Mucinous adenocarcinoma | female | white | alive | -- | stage iia | 22265 | Hepatic flexure of colon |
| TCGA-DM-A285 | TCGA-COAD | Mucinous adenocarcinoma | female | white | dead | 179 | stage iv | 26021 | Ascending colon |
| TCGA-DM-A288 | TCGA-COAD | Mucinous adenocarcinoma | male | white | dead | 427 | stage iiic | 25084 | Cecum |
| TCGA-DM-A28A | TCGA-COAD | Adenocarcinoma, NOS | male | white | dead | 805 | stage iiic | 28833 | Cecum |
| TCGA-DM-A28C | TCGA-COAD | Adenocarcinoma, NOS | male | white | dead | 2475 | stage iia | 27073 | Sigmoid colon |
| TCGA-DM-A28E | TCGA-COAD | Adenocarcinoma, NOS | female | white | alive | -- | stage iia | 26602 | Sigmoid colon |
| TCGA-DM-A28F | TCGA-COAD | Adenocarcinoma, NOS | male | white | dead | 1094 | stage iiib | 26856 | Sigmoid colon |
| TCGA-DM-A28G | TCGA-COAD | Adenocarcinoma, NOS | male | white | dead | 1849 | stage iia | 27641 | Ascending colon |
| TCGA-DM-A28H | TCGA-COAD | Adenocarcinoma, NOS | male | white | alive | -- | stage iiic | 18325 | Cecum |
| TCGA-DM-A28K | TCGA-COAD | Mucinous adenocarcinoma | male | white | alive | -- | stage iia | 27428 | Hepatic flexure of colon |
| TCGA-DM-A28M | TCGA-COAD | Adenocarcinoma, NOS | male | white | alive | -- | stage iia | 23043 | Descending colon |
| TCGA-DT-5265 | TCGA-READ | Mucinous adenocarcinoma | male | asian | alive | -- | stage ii | 18669 | Rectum, NOS |
| TCGA-DY-A0XA | TCGA-READ | Adenocarcinoma, NOS | female | white | alive | -- | stage iia | 21115 | Rectosigmoid junction |
| TCGA-DY-A1DC | TCGA-READ | Adenocarcinoma, NOS | female | white | dead | 1258 | stage iia | 26436 | Rectosigmoid junction |
| TCGA-DY-A1DD | TCGA-READ | Adenocarcinoma, NOS | female | white | dead | 1741 | stage iiib | 28159 | Rectosigmoid junction |
| TCGA-DY-A1DF | TCGA-READ | Adenocarcinoma, NOS | female | white | dead | 734 | stage iiic | 26763 | Rectosigmoid junction |
| TCGA-DY-A1DG | TCGA-READ | Adenocarcinoma, NOS | male | white | dead | 1566 | stage iva | 27396 | Rectum, NOS |
| TCGA-DY-A1H8 | TCGA-READ | Adenocarcinoma, NOS | female | white | dead | 992 | stage iiia | 28459 | Rectum, NOS |
| TCGA-EF-5830 | TCGA-READ | Adenocarcinoma, NOS | male | white | alive | -- | stage iib | 19802 | Rectum, NOS |
| TCGA-EF-5831 | TCGA-READ | Adenocarcinoma, NOS | male | white | alive | -- | stage iia | 26331 | Rectum, NOS |
| TCGA-EI-6506 | TCGA-READ | Tubular adenocarcinoma | female | white | alive | -- | stage iia | 28587 | Rectum, NOS |
| TCGA-EI-6507 | TCGA-READ | Mucinous adenocarcinoma | male | white | alive | -- | stage iia | 22176 | Rectum, NOS |
| TCGA-EI-6508 | TCGA-READ | Adenocarcinoma in tubolovillous adenoma | female | white | alive | -- | stage iiib | 17827 | Rectum, NOS |
| TCGA-EI-6509 | TCGA-READ | Adenocarcinoma in tubolovillous adenoma | male | white | alive | -- | stage iiic | 19666 | Rectum, NOS |
| TCGA-EI-6510 | TCGA-READ | Adenocarcinoma in tubolovillous adenoma | female | white | alive | -- | not reported | 28328 | Rectum, NOS |
| TCGA-EI-6511 | TCGA-READ | Adenocarcinoma in tubolovillous adenoma | male | white | alive | -- | stage iiib | 19237 | Rectum, NOS |
| TCGA-EI-6512 | TCGA-READ | Adenocarcinoma in tubolovillous adenoma | female | white | alive | -- | stage iiib | 23537 | Rectum, NOS |
| TCGA-EI-6513 | TCGA-READ | Adenocarcinoma in tubolovillous adenoma | male | white | alive | -- | stage iiib | 21776 | Rectum, NOS |
| TCGA-EI-6514 | TCGA-READ | Adenocarcinoma in tubolovillous adenoma | female | white | alive | -- | stage iia | 21618 | Rectum, NOS |
| TCGA-EI-6881 | TCGA-READ | Adenocarcinoma in tubolovillous adenoma | male | white | alive | -- | stage iiia | 22057 | Rectum, NOS |
| TCGA-EI-6882 | TCGA-READ | Adenocarcinoma with mixed subtypes | male | white | alive | -- | stage iia | 21682 | Rectum, NOS |
| TCGA-EI-6883 | TCGA-READ | Tubular adenocarcinoma | male | white | alive | -- | stage iic | 23268 | Rectum, NOS |
| TCGA-EI-6884 | TCGA-READ | Tubular adenocarcinoma | male | white | alive | -- | stage iiia | 25995 | Rectum, NOS |
| TCGA-EI-6885 | TCGA-READ | Tubular adenocarcinoma | female | white | alive | -- | stage iv | 21140 | Rectum, NOS |
| TCGA-EI-6917 | TCGA-READ | Adenocarcinoma with mixed subtypes | male | white | alive | -- | stage iiia | 12113 | Rectum, NOS |
| TCGA-EI-7002 | TCGA-READ | Tubular adenocarcinoma | male | white | alive | -- | stage iv | 21192 | Rectum, NOS |
| TCGA-EI-7004 | TCGA-READ | Mucinous adenocarcinoma | female | white | alive | -- | not reported | 13566 | Rectosigmoid junction |
| TCGA-F4-6459 | TCGA-COAD | Adenocarcinoma, NOS | female | white | dead | 262 | stage iiib | 22574 | Sigmoid colon |
| TCGA-F4-6460 | TCGA-COAD | Adenocarcinoma, NOS | female | white | dead | 972 | stage iiib | 18637 | Sigmoid colon |
| TCGA-F4-6461 | TCGA-COAD | Adenocarcinoma, NOS | female | white | dead | 338 | stage iiic | 15151 | Colon, NOS |
| TCGA-F4-6463 | TCGA-COAD | Mucinous adenocarcinoma | male | white | alive | -- | stage iia | 18804 | Transverse colon |
| TCGA-F4-6569 | TCGA-COAD | Adenocarcinoma, NOS | male | white | alive | -- | stage i | 22203 | Colon, NOS |
| TCGA-F4-6570 | TCGA-COAD | Adenocarcinoma, NOS | female | white | dead | 188 | stage iia | 28772 | Colon, NOS |
| TCGA-F4-6703 | TCGA-COAD | Adenocarcinoma, NOS | male | white | alive | -- | stage iia | 23644 | Ascending colon |
| TCGA-F4-6704 | TCGA-COAD | Mucinous adenocarcinoma | male | american indian or alaska native | alive | -- | stage iiic | 21949 | Sigmoid colon |
| TCGA-F4-6805 | TCGA-COAD | Adenocarcinoma, NOS | female | white | alive | -- | stage iia | 21241 | Colon, NOS |
| TCGA-F4-6806 | TCGA-COAD | Adenocarcinoma, NOS | female | white | alive | -- | stage i | 21625 | Sigmoid colon |
| TCGA-F4-6807 | TCGA-COAD | Adenocarcinoma, NOS | female | white | alive | -- | stage iiic | 18690 | Colon, NOS |
| TCGA-F4-6808 | TCGA-COAD | Adenocarcinoma, NOS | female | white | alive | -- | stage i | 19803 | Sigmoid colon |
| TCGA-F4-6809 | TCGA-COAD | Adenocarcinoma, NOS | female | white | dead | 403 | stage iva | 19039 | Sigmoid colon |
| TCGA-F4-6854 | TCGA-COAD | Adenocarcinoma, NOS | female | white | alive | -- | stage iia | 28272 | Sigmoid colon |
| TCGA-F4-6855 | TCGA-COAD | Adenocarcinoma, NOS | female | white | alive | -- | stage iia | 25890 | Sigmoid colon |
| TCGA-F4-6856 | TCGA-COAD | Mucinous adenocarcinoma | male | white | alive | -- | stage i | 16519 | Cecum |
| TCGA-F5-6464 | TCGA-READ | Adenocarcinoma, NOS | female | white | dead | 303 | stage iiic | 28168 | Rectosigmoid junction |
| TCGA-F5-6465 | TCGA-READ | Adenocarcinoma, NOS | female | white | alive | -- | stage iia | 23432 | Rectosigmoid junction |
| TCGA-F5-6571 | TCGA-READ | Adenocarcinoma, NOS | female | white | alive | -- | stage iia | 22666 | Rectum, NOS |
| TCGA-F5-6702 | TCGA-READ | Adenocarcinoma, NOS | male | white | alive | 869 | stage iva | 26123 | Rectosigmoid junction |
| TCGA-F5-6811 | TCGA-READ | Adenocarcinoma, NOS | female | white | alive | -- | stage iia | 26575 | Rectosigmoid junction |
| TCGA-F5-6814 | TCGA-READ | Adenocarcinoma, NOS | male | white | alive | -- | stage iia | 20828 | Rectum, NOS |
| TCGA-F5-6861 | TCGA-READ | Adenocarcinoma, NOS | female | white | alive | -- | stage iia | 21936 | Rectum, NOS |
| TCGA-F5-6863 | TCGA-READ | Adenocarcinoma, NOS | female | white | dead | 361 | stage iiib | 25936 | Rectum, NOS |
| TCGA-F5-6864 | TCGA-READ | Adenocarcinoma, NOS | female | white | alive | -- | stage iiib | 27058 | Rectum, NOS |
| TCGA-G4-6293 | TCGA-COAD | Adenocarcinoma, NOS | female | white | alive | -- | stage iii | 18076 | Transverse colon |
| TCGA-G4-6294 | TCGA-COAD | Adenocarcinoma, NOS | male | white | dead | 858 | stage iv | 27755 | Cecum |
| TCGA-G4-6295 | TCGA-COAD | Adenocarcinoma, NOS | female | white | alive | -- | stage ii | 25758 | Cecum |
| TCGA-G4-6297 | TCGA-COAD | Adenocarcinoma, NOS | female | white | alive | -- | stage iv | 20103 | Cecum |
| TCGA-G4-6298 | TCGA-COAD | Adenocarcinoma, NOS | male | black or african american | dead | 715 | stage iiib | 32872 | Cecum |
| TCGA-G4-6299 | TCGA-COAD | Adenocarcinoma, NOS | male | white | alive | -- | stage iiic | 25318 | Descending colon |
| TCGA-G4-6302 | TCGA-COAD | Mucinous adenocarcinoma | female | white | dead | 2047 | stage iia | 32872 | Cecum |
| TCGA-G4-6303 | TCGA-COAD | Adenocarcinoma, NOS | female | white | dead | 2003 | stage iv | 19800 | Sigmoid colon |
| TCGA-G4-6304 | TCGA-COAD | Adenocarcinoma, NOS | female | white | alive | -- | stage iib | 24172 | Transverse colon |
| TCGA-G4-6306 | TCGA-COAD | Adenocarcinoma, NOS | male | white | alive | -- | not reported | 26163 | Ascending colon |
| TCGA-G4-6307 | TCGA-COAD | Adenocarcinoma, NOS | female | white | alive | -- | stage iiib | 13648 | Sigmoid colon |
| TCGA-G4-6309 | TCGA-COAD | Adenocarcinoma, NOS | female | black or african american | alive | -- | stage iiib | 14729 | Sigmoid colon |
| TCGA-G4-6310 | TCGA-COAD | Adenocarcinoma, NOS | male | white | alive | -- | stage iiib | 25243 | Colon, NOS |
| TCGA-G4-6311 | TCGA-COAD | Adenocarcinoma, NOS | male | white | alive | -- | stage iii | 29297 | Ascending colon |
| TCGA-G4-6314 | TCGA-COAD | Adenocarcinoma, NOS | female | white | alive | -- | stage iv | 27958 | Cecum |
| TCGA-G4-6315 | TCGA-COAD | Adenocarcinoma, NOS | male | white | alive | -- | stage iv | 24174 | Descending colon |
| TCGA-G4-6317 | TCGA-COAD | Adenocarcinoma, NOS | female | white | alive | -- | stage iiic | 18822 | Sigmoid colon |
| TCGA-G4-6320 | TCGA-COAD | Adenocarcinoma, NOS | male | white | alive | -- | stage iii | 26840 | Hepatic flexure of colon |
| TCGA-G4-6321 | TCGA-COAD | Adenocarcinoma, NOS | female | white | alive | -- | stage iii | 21921 | Cecum |
| TCGA-G4-6322 | TCGA-COAD | Mucinous adenocarcinoma | male | white | alive | -- | stage iiib | 23825 | Descending colon |
| TCGA-G4-6323 | TCGA-COAD | Adenocarcinoma, NOS | male | black or african american | alive | -- | stage ia | 18334 | Cecum |
| TCGA-G4-6586 | TCGA-COAD | Adenocarcinoma, NOS | female | white | alive | -- | stage iia | 27020 | Ascending colon |
| TCGA-G4-6588 | TCGA-COAD | Adenocarcinoma, NOS | female | white | alive | -- | stage iia | 21532 | Cecum |
| TCGA-G4-6625 | TCGA-COAD | Adenocarcinoma, NOS | female | white | alive | -- | stage iia | 28163 | Sigmoid colon |
| TCGA-G4-6626 | TCGA-COAD | Adenocarcinoma, NOS | male | white | dead | 1422 | stage iia | 32871 | Ascending colon |
| TCGA-G4-6627 | TCGA-COAD | Adenocarcinoma, NOS | male | white | alive | -- | stage iia | 30697 | Ascending colon |
| TCGA-G4-6628 | TCGA-COAD | Adenocarcinoma, NOS | male | white | alive | -- | stage i | 28772 | Cecum |
| TCGA-G5-6233 | TCGA-READ | Adenocarcinoma, NOS | male | white | dead | 556 | not reported | 27190 | Rectum, NOS |
| TCGA-G5-6235 | TCGA-READ | Adenocarcinoma, NOS | male | white | alive | -- | stage iiib | 26348 | Rectosigmoid junction |
| TCGA-G5-6572 | TCGA-READ | Adenocarcinoma, NOS | male | white | dead | 1432 | not reported | 20475 | Rectosigmoid junction |
| TCGA-G5-6641 | TCGA-READ | Mucinous adenocarcinoma | male | white | alive | -- | stage iiia | 24486 | Rectosigmoid junction |
| TCGA-NH-A50T | TCGA-COAD | Adenocarcinoma, NOS | female | black or african american | alive | -- | stage iia | 25089 | Splenic flexure of colon |
| TCGA-NH-A50U | TCGA-COAD | Mucinous adenocarcinoma | male | black or african american | dead | 334 | stage iva | 15450 | Cecum |
| TCGA-NH-A50V | TCGA-COAD | Adenocarcinoma, NOS | male | black or african american | alive | -- | stage iiib | 25467 | Cecum |
| TCGA-NH-A5IV | TCGA-COAD | Adenocarcinoma, NOS | female | black or african american | alive | -- | stage iia | 32872 | Transverse colon |
| TCGA-NH-A6GA | TCGA-COAD | Adenocarcinoma, NOS | male | black or african american | dead | 302 | stage iiic | 21313 | Ascending colon |
| TCGA-NH-A6GB | TCGA-COAD | Adenocarcinoma, NOS | female | black or african american | alive | -- | stage iiic | 26100 | Transverse colon |
| TCGA-NH-A6GC | TCGA-COAD | Mucinous adenocarcinoma | female | black or african american | alive | -- | stage ivb | 24355 | Descending colon |
| TCGA-NH-A8F7 | TCGA-COAD | Adenocarcinoma, NOS | female | black or african american | alive | -- | stage iia | 19535 | Sigmoid colon |
| TCGA-NH-A8F8 | TCGA-COAD | Adenocarcinoma, NOS | male | black or african american | dead | 511 | stage iv | 29185 | Ascending colon |
| TCGA-QG-A5YV | TCGA-COAD | Adenocarcinoma, NOS | female | black or african american | alive | -- | stage iiic | 23499 | Sigmoid colon |
| TCGA-QG-A5YW | TCGA-COAD | Adenocarcinoma, NOS | female | black or african american | alive | -- | stage iiic | 20391 | Cecum |
| TCGA-QG-A5YX | TCGA-COAD | Adenocarcinoma, NOS | female | black or african american | alive | -- | stage iia | 22617 | Sigmoid colon |
| TCGA-QG-A5Z1 | TCGA-COAD | Adenocarcinoma, NOS | male | black or african american | dead | 256 | stage iiib | 26220 | Rectosigmoid junction |
| TCGA-QG-A5Z2 | TCGA-COAD | Adenocarcinoma, NOS | male | black or african american | alive | -- | stage i | 22367 | Cecum |
| TCGA-QL-A97D | TCGA-COAD | Adenocarcinoma, NOS | female | black or african american | alive | -- | stage i | 30914 | Cecum |
| TCGA-RU-A8FL | TCGA-COAD | Adenocarcinoma, NOS | male | black or african american | alive | -- | stage iiib | 18975 | Cecum |
| TCGA-SS-A7HO | TCGA-COAD | Adenocarcinoma, NOS | female | black or african american | alive | -- | stage iib | 16416 | Cecum |
| TCGA-T9-A92H | TCGA-COAD | Adenocarcinoma, NOS | male | black or african american | alive | -- | stage iia | 30058 | Sigmoid colon |
| TCGA-WS-AB45 | TCGA-COAD | Mucinous adenocarcinoma | female | black or african american | alive | -- | stage iia | -- | Cecum |

**Supplementary Table S3. The distribution of the 44 mutated genes in different pathways with more than 30 scores based on the GeneCards database**

| Gene | Score | Frequency-TCGA (%) | Frequency-validation (%) | |
| --- | --- | --- | --- | --- |
| **Wnt signaling** | | | | |
| CTNNB1 | 99.73 | 7.16 | 5.66 | |
| APC | 56.31 | 76.65 | 60.38 | |
| TP53 | 50.37 | 59.70 | 64.15 | |
| MTOR | 43.60 | 7.53 | 15.09 | |
| PIK3CA | 41.12 | 25.24 | 16.98 | |
| PTEN | 39.37 | 6.78 | 20.75 | |
| TCF7L2 | 36.49 | 8.85 | 7.55 | |
| SMAD4 | 36.34 | 12.99 | 15.09 | |
| KRAS | 32.28 | 40.49 | 47.17 | |
| AXIN2 | 31.90 | 6.21 | 5.66 | |
| **RAS signaling** | | | | |
| KRAS | 94.42 | 40.49 | 47.17 | |
| NRAS | 72.56 | 6.21 | 13.21 | |
| PIK3CA | 66.14 | 25.24 | 16.98 | |
| TP53 | 66.06 | 59.70 | 64.15 | |
| BRAF | 64.76 | 12.62 | 16.98 | |
| CTNNB1 | 55.67 | 7.16 | 5.66 | |
| MTOR | 51.59 | 7.53 | 15.09 | |
| NF1 | 50.85 | 6.40 | 22.64 | |
| PTEN | 43.53 | 6.78 | 20.75 | |
| PIK3R1 | 42.19 | 5.46 | 7.55 | |
| SMAD4 | 38.18 | 12.99 | 15.09 | |
| RET | 33.68 | 5.27 | 15.09 | |
| APC | 31.06 | 76.65 | 60.38 | |
| KDR | 30.26 | 5.27 | 15.09 | |
| **TGF-beta signaling** | | | | |
| SMAD4 | 82.81 | 12.99 | 15.09 | |
| CTNNB1 | 44.72 | 7.16 | 5.66 | |
| TP53 | 42.53 | 59.70 | 64.15 | |
| PIK3CA | 37.91 | 25.24 | 16.98 | |
| KRAS | 36.36 | 40.49 | 47.17 | |
| PTEN | 33.57 | 6.78 | 20.75 | |
| **p53 signaling** | | | | |
| TP53 | 167.33 | 59.70 | 64.15 | |
| PTEN | 55.15 | 6.78 | 20.75 | |
| CTNNB1 | 54.65 | 7.16 | 5.66 | |
| PIK3CA | 49.11 | 25.24 | 16.98 | |
| ATM | 45.99 | 13.56 | 28.30 | |
| MTOR | 45.12 | 7.53 | 15.09 | |
| KRAS | 43.02 | 40.49 | 47.17 | |
| BRAF | 39.42 | 12.62 | 16.98 | |
| EP300 | 38.81 | 5.46 | 9.43 | |
| SMAD4 | 38.47 | 12.99 | 15.09 | |
| PIK3R1 | 35.35 | 5.46 | 7.55 | |
| APC | 34.64 | 76.65 | 60.38 | |
| CREBBP | 31.92 | 8.85 | 7.55 | |
| BRCA2 | 30.62 | 8.47 | 16.98 | |
| **PI3K-Akt signaling** | | | | |
| PIK3CA | 77.66 | 25.24 | 16.98 | |
| PTEN | 76.58 | 6.78 | 20.75 | |
| MTOR | 66.18 | 7.53 | 15.09 | |
| TP53 | 54.37 | 59.70 | 64.15 | |
| CTNNB1 | 52.87 | 7.16 | 5.66 | |
| PIK3R1 | 48.67 | 5.46 | 7.55 | |
| KRAS | 44.34 | 40.49 | 47.17 | |
| BRAF | 40.96 | 12.62 | 16.98 | |
| NRAS | 39.36 | 6.21 | 13.21 | |
| SMAD4 | 31.44 | 12.99 | 15.09 | |
| APC | 30.21 | 76.65 | 60.38 | |
| **MAPK signaling** | | | | |
| PIK3CA | 49.74 | 25.24 | 16.98 | |
| BRAF | 49.73 | 12.62 | 16.98 | |
| TP53 | 46.42 | 59.70 | 64.15 | |
| CTNNB1 | 46.30 | 7.16 | 5.66 | |
| MTOR | 44.47 | 7.53 | 15.09 | |
| KRAS | 43.44 | 40.49 | 47.17 | |
| PIK3R1 | 39.17 | 5.46 | 7.55 | |
| PTEN | 35.82 | 6.78 | 20.75 | |
| NF1 | 35.57 | 6.40 | 22.64 | |
| NRAS | 35.11 | 6.21 | 13.21 | |
