# Supplementary figures and images for: Multi gene mutation signatures in colorectal cancer patients: predict for the diagnosis, pathological classification, staging and prognosis
Source: BMC Cancer. 2021 Apr 9;21:380. doi: 10.1186/s12885-021-08108-9 (PMC8034139; doi:10.1186/s12885-021-08108-9)

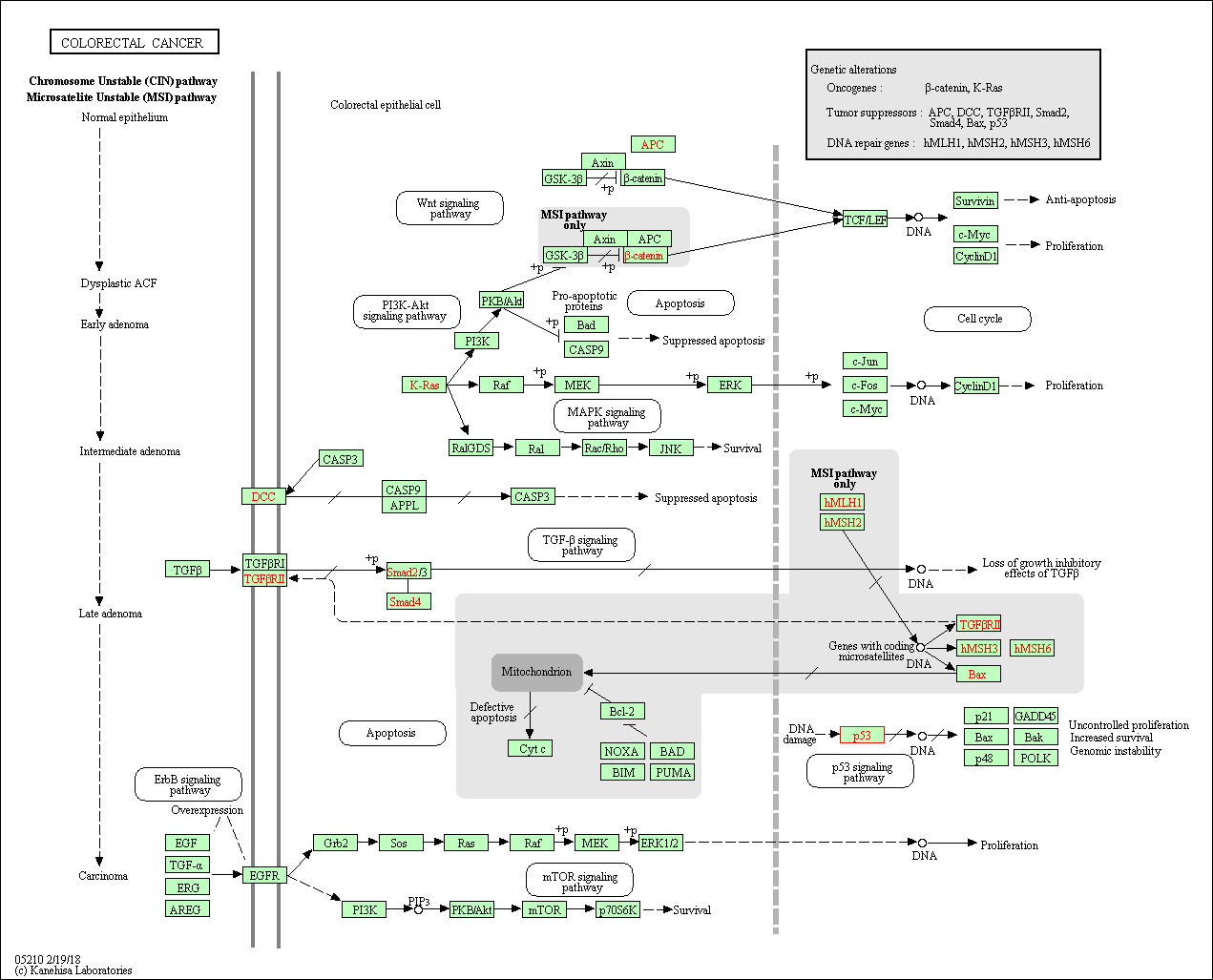

Supplement: Supplementary file 2 — Additional file 2. [file 12885_2021_8108_MOESM2_ESM.png]
